# Effects of transcranial direct current stimulation on gait and balance under dual-task conditions in older adults: a systematic review and meta-analysis

**DOI:** 10.3389/fnagi.2026.1924261

**Published:** 2026-09-28

**Authors:** Yunxing Bi, Hongjie Ni, Ziqing Hou, Jinghua Qian

**Affiliations:** 1 School of Sports Medicine and Rehabilitation, Beijing Sport University, Beijing, China; 2 Key Laboratory of Exercise Rehabilitation Science of the Ministry of Education, Beijing Sport University, Beijing, China

**Keywords:** balance, dual-task cost, dual-task gait, dual-task performance, meta-analysis, older adults, systematic review, transcranial direct current stimulation

## Abstract

**Objective:**

This systematic review and meta-analysis evaluated the effects of transcranial direct current stimulation (tDCS) on gait and balance outcomes measured under dual-task (DT) conditions in older adults.

**Methods:**

Following PRISMA guidance, four databases were searched through October 13, 2025. Random-effects meta-analyses used Hedges’ *g*. Healthy older adults were prespecified as the primary population; clinical populations were analysed separately and mixed-population estimates were treated as exploratory. Multi-arm estimates were consolidated at the study level. Crossover trials used an assumed within-participant correlation (corr) of 0.50, with sensitivity analyses at 0.25 and 0.75. Risk of bias was assessed with RoB 2 and certainty with GRADE.

**Results:**

Fifteen studies involving 385 participants were included, and 12 contributed to quantitative synthesis. The forest plots present exploratory mixed-population estimates: dual-task gait speed was not significantly improved (Hedges’ *g* = 0.071, 95% CI -0.199 to 0.340; *p* = 0.607), whereas the dual-task cost (DTC) of gait speed was reduced (Hedges’ *g* = −0.362, 95% CI -0.538 to −0.185; *p* < 0.001). Exploratory reductions were also observed for DTC of sway speed, sway area, and DTC of sway area. In the prespecified healthy-older-adult primary analysis, gait speed (Hedges’ *g* = 0.200, 95% CI 0.017 to 0.383; *p* = 0.032) and its DTC (Hedges’ *g* = −0.368, 95% CI -0.557 to −0.179; *p* < 0.001) favoured tDCS. The gait-speed result was sensitive to the assumed crossover correlation, whereas gait-speed DTC remained significant across correlation assumptions.

**Conclusion:**

In healthy older adults, tDCS may improve gait speed and reduce its dual-task cost, but the gait-speed inference depends on the assumed crossover correlation and both estimates are based on only two studies. Evidence for other gait, variability, mobility, and standing-balance outcomes was inconclusive or could not be pooled within the healthy population. Evidence regarding falls, long-term benefit, and safety remains insufficient.

**Systematic review registration:**

https://www.crd.york.ac.uk/PROSPERO/view/CRD420251206965, PROSPERO Unique Identifier: CRD420251206965.

## 1 Introduction

A fall refers to an event when a person accidentally lands on the ground, floor, or other surface below their original position (World Health Organization, 2021). With the increasing aging of the global population, falls in older adults have become an important public health issue. According to WHO data, about 684,000 people worldwide die from fall-related injuries each year, with adults aged 60 and above accounting for the largest proportion, resulting in substantial economic costs (World Health Organization, 2021). However, falls in older adults are not simply caused by “physical instability.” Besides factors such as age, frailty, comorbidity, gait, muscle strength, and balance control (Beck Jepsen et al., 2022), falls are also closely related to the ability to handle “multiple tasks” in daily living.

In daily life, a dual task (DT) occurs when an individual performs two or more tasks simultaneously, such as walking while using a mobile phone. Performance under these conditions is referred to as dual-task performance (DTP) (Watanabe and Funahashi, 2018) According to bottleneck and capacity-sharing theories, concurrent tasks compete for limited processing capacity and attentional resources (Tsang et al., 2022). When the demands of two tasks exceed the available processing capacity, performance on one or both tasks may decline. This phenomenon is known as dual-task interference (DTI), and it is commonly quantified using the dual-task cost (DTC) (Paas Oliveros et al., 2023; Zhou et al., 2026). Since postural control is governed by a nonlinear dynamic system, recent studies have increasingly employed nonlinear analyses of the postural sway, such as approximate entropy, Lyapunov exponent, correlation dimension, and detrended fluctuation analysis, which provide additional insights into motor-cognitive interference beyond traditional linear measures (Saraiva et al., 2022; Saraiva et al., 2023). For instance, a nonlinear study examining the effect of different types of cognitive tasks on postural sway fluctuations in older and younger adults revealed that cognitive dual-tasking induces sway impairments and lower postural control efficacy (Rafiee Manesh et al., 2024). Neuroimaging studies have shown selective recruitment of the prefrontal cortex (PFC), a region involved in executive function and working memory, during DT performance (Miura et al., 2024). Older adults commonly show greater PFC activation than younger adults, suggesting increased reliance on compensatory executive control (Watanabe and Funahashi, 2018; Miura et al., 2024). Ageing is also accompanied by pronounced structural and functional brain changes. Structurally, aging is associated with widespread brain atrophy, with the most pronounced volumetric and cortical changes observed in sensorimotor and frontoparietal regions, alongside white-matter degeneration and reduced myelination (Hupfeld et al., 2022; Koppelmans et al., 2017). These structural alterations extend beyond global atrophy: multimodal neuroimaging evidence indicates that regional cortical thinning and altered white-matter microstructure in older adults are specifically related to greater dual-task cost of gait speed and variability (Koppelmans et al., 2017). Functionally, aging is marked by impaired motor preparation and reduced cortical readiness during voluntary motor tasks. For example, using both neurophysiological and biomechanical measures, Khanmohammadi et al. (2015) demonstrated that preparatory postural adjustments during gait initiation are impaired in healthy older compared with younger adults. Transcranial magnetic stimulation studies show that anticipatory postural adjustments of the trunk and hip musculature are delayed in older adults and are accompanied by altered spatial organization of trunk/hip muscle representations in the motor cortex (Rowland et al., 2021; Smith and Fisher, 2018), suggesting that age-related decline in anticipatory motor control has both peripheral and cortical determinants. These changes may constrain flexible allocation of cognitive and motor resources. Impaired DT gait and balance have been associated with increased fall risk in older adults (Beck Jepsen et al., 2022; Barolo et al., 2026; Beauchet et al., 2009; Beurskens and Bock, 2012; Lara et al., 2024; Ramírez and Gutiérrez, 2021); falls may in turn result in fractures, head injury, loss of independence, and mortality (World Health Organization, 2021). These observations provide a rationale for examining whether neuromodulation can improve gait and balance under DT conditions.

Transcranial direct current stimulation (tDCS) is a non-invasive neuromodulation technique that delivers weak direct current, typically 1–2 mA, to targeted cortical regions through scalp electrodes (Lefaucheur et al., 2017). By modulating neuronal membrane potential, tDCS can alter cortical excitability and plasticity (Ljubisavljevic et al., 2019; Nitsche and Paulus, 2000) Anodal stimulation of the left dorsolateral prefrontal cortex (DLPFC), primary motor cortex (M1), or related networks has been investigated as a means of modifying motor-cognitive interference during walking and standing (Schneider et al., 2021; Zhou et al., 2015; Zhou et al., 2021). Potential effects may differ by stimulation target: DLPFC stimulation may influence executive control, attention allocation, and working memory; M1 stimulation may influence motor preparation and output; and cerebellar stimulation may influence timing, prediction, and sensorimotor integration (Khanmohammadi et al., 2025a). Because the available quantitative evidence mainly concerns motor outcomes rather than concurrent cognitive-task performance, the present review evaluates gait and balance outcomes measured under DT conditions and treats broader DTP claims cautiously.

## 2 Methods

### 2.1 Study design

This systematic review was reported following the Preferred Reporting Items for Systematic Reviews and Meta-Analyses (PRISMA) guidelines (Page et al., 2020; Page et al., 2021). Before conducting the literature search, the study protocol was prospectively registered in the International Prospective Register of Systematic Reviews (PROSPERO) under the registration number CRD420251206965.

### 2.2 Literature search

A search strategy based on the PICOS framework (Population, Intervention, Comparison, Outcome, and Study design) combined Medical Subject Headings and free-text terms. PubMed, Web of Science, Embase, and the Cochrane Library were searched from inception to October 13, 2025. The complete database-specific search strategies, platforms, fields, and limits are provided in Supplementary Appendix 1.

### 2.3 Inclusion and exclusion criteria

Eligibility criteria were defined using PICOS. The primary population was healthy or community-dwelling older adults aged ≥60 years or samples with a mean age ≥65 years. Clinical populations—including mild cognitive impairment, Parkinson’s disease, functional limitation, and knee osteoarthritis—were eligible for the systematic review but were not combined with healthy participants in the primary meta-analysis. Each clinical population was synthesized separately when at least two sufficiently comparable studies were available; otherwise, findings were reported narratively. Studies were excluded when participants had severe neurological or musculoskeletal disability that precluded ambulatory DT testing, e.g., PD (Parkinson’s disease) Hoehn-Yahr stage >III, wheelchair dependence, functionally dependent multiple sclerosis, post-stroke hemiplegia, severe arthritis, or limb amputation; when the study was non-randomized or lacked a sham comparator; when no DT gait, mobility, or balance outcome was reported; when usable data or full text could not be obtained; when the report was not in English; or when it was a duplicate, protocol, abstract, conference proceeding, dissertation, letter, or guideline.

### 2.4 Data extraction

Two reviewers independently extracted study characteristics and outcome data using a standardized form, with disagreements resolved by a third reviewer. Extracted items included study design, population, sample size, age and sex, tDCS polarity, montage, electrode size, current intensity, duration, number of sessions, DT paradigm, task-priority instructions, assessment time point, gait and balance outcomes, concurrent cognitive-task outcomes, adverse events, and follow-up. For each quantitative comparison, we recorded the active and comparator arms, whether final or change scores were used, the DTC formula and beneficial direction, and any sign transformation. Change-score standard deviations were calculated according to the Cochrane Handbook when not reported. The prespecified correlation between pre- and post-intervention measurements was 0.50, and sensitivity analyses used correlations of 0.25 and 0.75.

$$SD_{\text{change}}=\sqrt{SD_{\mathrm{pre}}^{2}+SD_{\text{post}}^{2}-(2\times \text{corr}\times SD_{\mathrm{pre}}\times SD_{\text{post}})}$$

In the formula, corr denotes the within-participant correlation between pre- and post-intervention measurements. A value of 0.50 was used in the primary calculation, with 0.25 and 0.75 examined in sensitivity analyses (Higgins et al., 2022). Corresponding authors were contacted at least three times when required data were unavailable. All extracted values and effect-direction transformations were independently verified by a third reviewer.

### 2.5 Quality assessment

Methodological quality was assessed with the Cochrane RoB 2 tool (Lundh and Gøtzsche, 2008). Parallel-group trials were evaluated across bias arising from the randomization process, deviations from intended interventions, missing outcome data, outcome measurement, and selection of the reported result. The crossover version additionally considered period and carryover effects. Two reviewers independently assessed risk of bias for the outcomes and time points contributing to the quantitative syntheses; disagreements were resolved by consensus or a third reviewer. Overall judgements were low risk, some concerns, or high risk.

### 2.6 Statistical analysis

Meta-analyses used Hedges’ g with 95% CIs. Healthy older adults constituted the primary analysis population. Clinical populations were analysed separately, and mixed-population pooling was retained only as an explicitly exploratory analysis in the Supplementary material. Change scores were preferred when available; final values were used when changes could not be derived. For crossover trials, paired standardized effects were calculated using corr = 0.50 when the reported within-participant correlation was unavailable (Stedman et al., 2011); corr = 0.25 and 0.75 were examined in sensitivity analyses. Multiple active conditions or task variants from the same participants were consolidated into one study-level estimate (assumed correlation between estimates = 0.50), preventing repeated use of the same sham condition. Random-effects models used the DerSimonian–Laird estimator. *I*^2^ and Cochran’s Q quantified heterogeneity (Higgins et al., 2003). Positive values favoured tDCS for gait speed, cadence, and stride length; negative values favoured tDCS for time, variability, sway, and harmonized DTC outcomes.

### 2.7 Reporting Bias and certainty of evidence

Small-study and publication bias were to be assessed using funnel plots and regression-based asymmetry tests only when an outcome included at least 10 studies. Because no pooled outcome met this threshold, publication bias could not be assessed reliably. Certainty of evidence for key outcomes was rated using GRADE across risk of bias, inconsistency, indirectness, imprecision, and publication bias. Two reviewers made independent judgements and resolved disagreements by consensus.

## 3 Results

### 3.1 Study selection

The literature search was completed on October 13, 2025 and identified 4,802 records: 370 from PubMed, 2,494 from Web of Science, 777 from Embase, and 1,161 from the Cochrane Library. After removal of 1,383 duplicates, 3,419 records underwent title and abstract screening, of which 3,293 were excluded. Full texts of 126 reports were assessed, and 111 were excluded for prespecified reasons. Fifteen studies were included in the qualitative synthesis, and 12 contributed to at least one meta-analysis (Figure 1).

**Figure 1:**
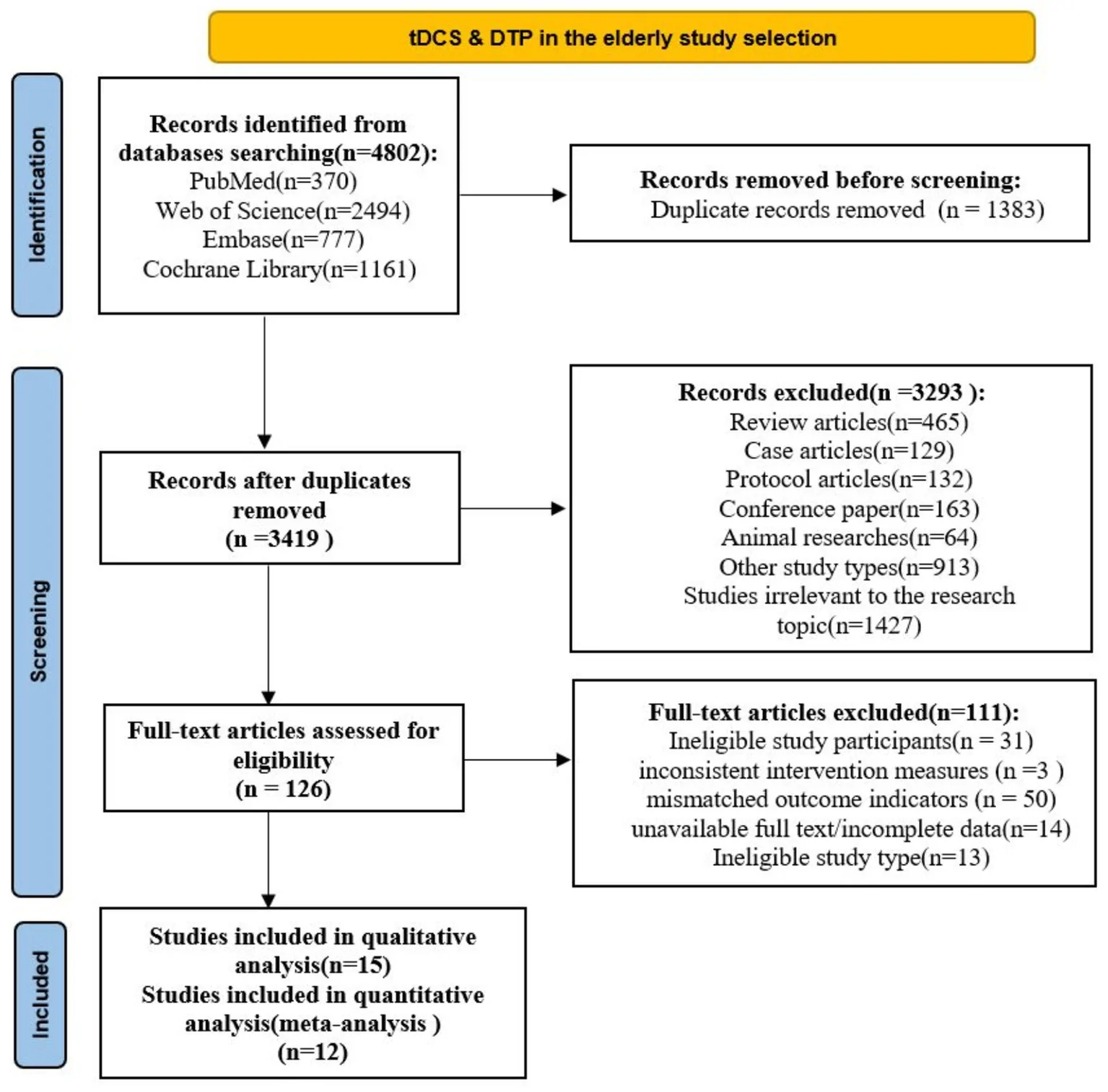
PRISMA 2020 flow diagram of study selection. Fifteen studies were included in the qualitative synthesis, and 12 contributed to at least one quantitative synthesis.

### 3.2 Quality assessment results

Risk of bias was assessed in 15 randomized trials, comprising eight parallel-group and seven crossover trials (Figure 2). Among the eight parallel RCTs, three (Lau et al., 2024; Liao et al., 2021; Manor et al., 2018) were judged to have low risk of bias overall, four (Jor'dan et al., 2022; Kalkhoran and Khanmohammadi, 2025; Schabrun et al., 2016; Orcioli-Silva et al., 2021) raised some concerns, and one (Rodrigues et al., 2023) was judged at high risk. For the domains of deviations from intended interventions (D2) and missing outcome data (D3), all studies were rated as low risk. For the randomization process (D1), one (Rodrigues et al., 2023) study was rated as high risk and one (Schabrun et al., 2016) raised some concerns, due to insufficient reporting of the random sequence generation or allocation concealment. For outcome measurement (D4), only one (Rodrigues et al., 2023) study raised some concerns, because of missing information on blinding of outcome assessment. For selective reporting of results (D5), three studies (Jor'dan et al., 2022; Kalkhoran and Khanmohammadi, 2025; Orcioli-Silva et al., 2021) raised some concerns.

**Figure 2:**
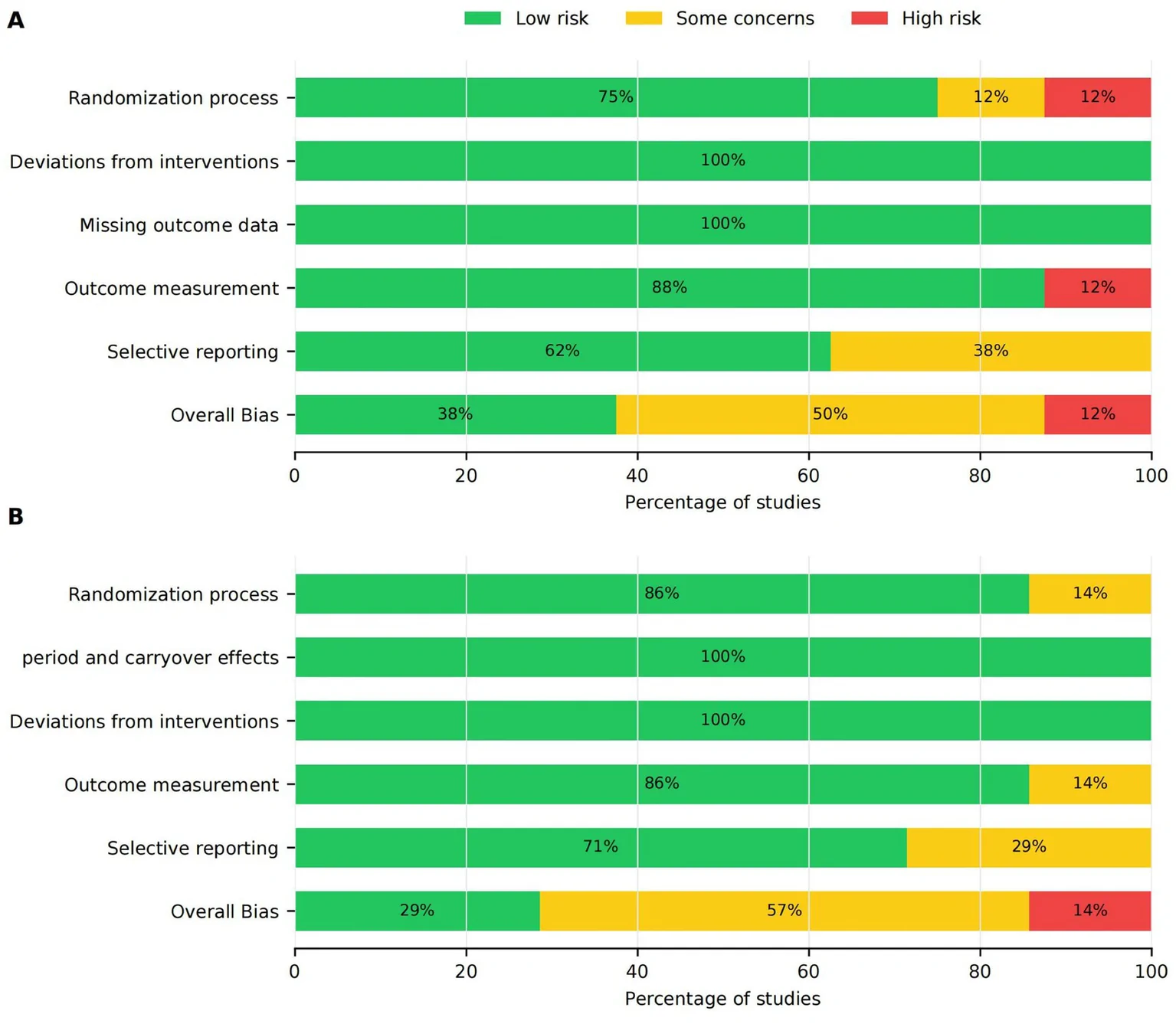
Risk-of-bias summary for **(A)** parallel-group and **(B)** crossover trials, showing the proportions judged as low risk, some concerns, or high risk across RoB 2 domains and overall. For crossover trials, period and carryover effects were additionally assessed.

Among the seven crossover RCTs, only two (Zhou et al., 2021; Baharlouei et al., 2020) were judged to be at low risk of bias overall, four (Khanmohammadi et al., 2025b; Mishra and Thrasher, 2022; Swank et al., 2016; Yun et al., 2025) raised some concerns, and one (Schneider et al., 2021) was at high risk. For the crossover-specific domain of period and carryover effects (DS) and the domain of deviations from intended interventions (D2), all studies were rated as low risk. For the randomization process (D1), only one (Swank et al., 2016) study raised some concerns. For missing outcome data (D3), one (Schneider et al., 2021) study was rated at high risk and two (Khanmohammadi et al., 2025b; Yun et al., 2025) raised some concerns, which were related to participant dropout across periods and insufficient reporting of how missing data were handled. For outcome measurement (D4), only one (Swank et al., 2016) study raised some concerns. For selective reporting of results (D5), two (Mishra and Thrasher, 2022; Swank et al., 2016) studies raised some concerns. Across the crossover evidence, incomplete handling of missing observations and limited reporting of period or carryover effects were the principal recurring concerns.

### 3.3 Participant characteristics

A total of 15 studies involving 385 older adults (260 females and 125 males) were included in this review. The studies were conducted across multiple countries, including the United States, Brazil, the United Kingdom, China, Iran, South Korea, and Australia. six of these studies included healthy older adults (Schneider et al., 2021; Zhou et al., 2021; Orcioli-Silva et al., 2021; Rodrigues et al., 2023; Baharlouei et al., 2020; Khanmohammadi et al., 2025b), three studies focused on patients with PD (stages I–III) (Schabrun et al., 2016; Mishra and Thrasher, 2022; Yun et al., 2025), two studies targeted older adults with mild cognitive impairment (Lau et al., 2024; Liao et al., 2021), two studies included older adults with functional limitations (Manor et al., 2018; Jor'dan et al., 2022), one study included older women with knee osteoarthritis (Kalkhoran and Khanmohammadi, 2025), and one study included patients with PD and mild motor and executive dysfunction (Swank et al., 2016). The overall age range of the participants was 65.17 to 80.50 years. Regarding cognitive function assessment, seven studies used the Montreal Cognitive Assessment (MoCA) (Zhou et al., 2021; Liao et al., 2021; Manor et al., 2018; Orcioli-Silva et al., 2021; Mishra and Thrasher, 2022; Yun et al., 2025), six studies used the Mini-Mental State Examination (MMSE) (Schneider et al., 2021; Lau et al., 2024; Jor'dan et al., 2022; Schabrun et al., 2016; Rodrigues et al., 2023; Khanmohammadi et al., 2025b), and the remaining three studies did not report the level of cognitive function (Kalkhoran and Khanmohammadi, 2025; Baharlouei et al., 2020; Swank et al., 2016). According to established criteria, MMSE scores > 24 and MoCA scores > 26 were considered to indicate normal cognitive function, suggesting that the overall cognitive function of most participants was within the normal or mildly impaired range (Folstein et al., 1975; Nasreddine et al., 2005). Detailed participant characteristics for each study are presented in Table 1.

**Table 1:** Participant characteristics of the included studies.

Study	Country	Design	Health status	Sample (n)	Age	Cognitive assessment
Zhou et al. (2021)	USA	Double-blinded, randomized, sham-controlled, cross-over	Healthy older adults	57 (43F,14M)	75.00 ± 4.96	MOCA:26.54 ± 3.02
Schneider et al. (2021)	USA	Double-blinded, randomized, sham-controlled, cross-over	Healthy older adults	25 (20F, 5M)	73.90 ± 5.20	MOCA:26.60 ± 2.80
Rodrigues et al. (2023)	Brazil.	Single-blinded, randomized controlled	Community-dwelling older adults	27 (18F, 9M)	71.11 ± 7.46	MMSE:26.88 ± 2.06
Orcioli-Silva et al. (2021)	UK	Double-blinded, randomized, sham-controlled	Healthy older adults	19 (12F, 7M)	67.85 ± 5.84	MOCA:28.25 ± 1.32
Manor et al. (2018)	USA	Sham-controlled, double-blinded, block-randomized	Functionally limited older adults	18 (10F, 8M)	80.50 ± 4.18	MOCA:22.00 ± 3.43
Liao et al. (2021)	China	Double-blinded, randomized controlled	Older adults with mild cognitive impairment	20 (13F, 7M)	72.85 ± 4.25	MOCA:23.75 ± 4.00
Lau et al. (2024)	China	Double-blinded, randomized controlled	Older adults with mild cognitive impairment	21 (14F, 7M)	70.57 ± 12.76	MMSE:25.37 ± 2.15
Khanmohammadi et al. (2025a)	Iran	Randomized, double-blind, crossover-controlled	Healthy older adults	19 (15F, 4M)	71.22 ± 5.82	MMSE:28.30 ± 0.78
Jor'dan et al. (2022)	USA	double-blinded, randomized, sham-controlled	Functionally limited older adults	14 (6F, 8M)	80.00 ± 11.00	MMSE:26.50 ± 4.00
Baharlouei et al. (2020)	Iran	Double-blinded, sham-controlled, cross-over	Healthy older adults	32 (16F, 16M)	67.60 ± 6.29	NR
Kalkhoran and Khanmohammadi (2025)	Iran	Randomized, triple-blind, sham-controlled	Elderly women with KOA	68 (68F, 0M)	65.17 ± 1.55	NR
Mishra and Thrasher (2022)	USA	Randomized, double-blind, cross-over, sham-controlled	Parkinson’s disease (I–III)	20 (6F, 14M)	67.80 ± 8.30	MOCA:26.1 ± 2.2
Yun et al. (2025)	Korea	Single-center, double-blind, explorative, pilot randomized crossover trial	Parkinson’s disease (I–III)	19 (11F, 8M)	72.79 ± 5.39	MOCA:24.00 ± 3.00
Swank et al. (2016)	USA	Randomized, single-blind, cross-over, sham-controlled trial	Parkinson’s disease	10 (2F, 8M)	68.70 ± 10.20	NR
Schabrun et al. (2016)	Australia	Randomized, double-blind, sham-controlled, parallel-group pilot trial	Parkinson’s Disease (II-III)	16 (6F, 10M)	67.50 ± 9.45	MMSE:29.35 ± 0.71

F, female; M, male; MMSE, mini-mental state exam; MoCA, montreal cognitive assessment; NR, not reported; Data for age and cognitive function levels are mean ± standard deviation; The MMSE and MoCA scores range from 0 to 30.

### 3.4 tDCS characteristics

Table 2 presents the detailed characteristics of the tDCS intervention. All studies applied sham stimulation as the control condition and employed anodal stimulation, with the anode placed over the target brain region to enhance cortical excitability. Three of these studies administered tDCS concurrently with the task (Schneider et al., 2021; Schabrun et al., 2016; Yun et al., 2025), while the remaining studies had participants perform the task either before or after tDCS stimulation. Regarding electrode configuration, electrode shapes included round electrodes (Schneider et al., 2021; Zhou et al., 2021; Khanmohammadi et al., 2025b; Yun et al., 2025), rectangular electrodes (Lau et al., 2024; Liao et al., 2021; Rodrigues et al., 2023; Baharlouei et al., 2020; Mishra and Thrasher, 2022), and square electrodes (Orcioli-Silva et al., 2021; Swank et al., 2016). Some studies employed conventional bipolar electrode configurations and their variants (Lau et al., 2024; Liao et al., 2021; Manor et al., 2018; Jor'dan et al., 2022; Kalkhoran and Khanmohammadi, 2025; Schabrun et al., 2016; Orcioli-Silva et al., 2021; Rodrigues et al., 2023; Baharlouei et al., 2020; Mishra and Thrasher, 2022; Yun et al., 2025), while others adopted a multi-target current-optimized layout (Schneider et al., 2021; Zhou et al., 2021). one study used high-definition tDCS with a 4 × 1 ring electrode array (Khanmohammadi et al., 2025b), and five studies did not specify the electrode shape (Manor et al., 2018; Jor'dan et al., 2022; Kalkhoran and Khanmohammadi, 2025; Schabrun et al., 2016; Swank et al., 2016). Regarding electrode placement, most studies placed the anode over the L-DLPFC region (Schneider et al., 2021; Zhou et al., 2021; Lau et al., 2024; Liao et al., 2021; Manor et al., 2018; Jor'dan et al., 2022; Orcioli-Silva et al., 2021; Rodrigues et al., 2023; Khanmohammadi et al., 2025b; Mishra and Thrasher, 2022; Swank et al., 2016; Yun et al., 2025), corresponding to the F3 site of the international 10–20 system, while a minority involved regions such as M1, the cerebellum, and the posterior parietal cortex (Schneider et al., 2021; Zhou et al., 2021; Schabrun et al., 2016; Orcioli-Silva et al., 2021; Baharlouei et al., 2020; Khanmohammadi et al., 2025b; Mishra and Thrasher, 2022; Yun et al., 2025). The cathode was typically placed above the contralateral supraorbital region or at other reference sites. The tDCS intervention protocols were classified into single-session stimulation (accounting for approximately 53.3% of studies) (Schneider et al., 2021; Zhou et al., 2021; Orcioli-Silva et al., 2021; Baharlouei et al., 2020; Khanmohammadi et al., 2025b; Mishra and Thrasher, 2022; Swank et al., 2016; Yun et al., 2025) and repeated multi-session stimulation (up to 36 sessions) (Lau et al., 2024; Liao et al., 2021; Manor et al., 2018; Jor'dan et al., 2022; Kalkhoran and Khanmohammadi, 2025; Schabrun et al., 2016; Rodrigues et al., 2023) Approximately 92.3% of the included studies selected a stimulation intensity of 2 mA (Lau et al., 2024; Liao et al., 2021; Manor et al., 2018; Jor'dan et al., 2022; Kalkhoran and Khanmohammadi, 2025; Schabrun et al., 2016; Rodrigues et al., 2023; Baharlouei et al., 2020; Khanmohammadi et al., 2025b; Mishra and Thrasher, 2022; Swank et al., 2016; Yun et al., 2025), while only three studies used lower intensities (0.5–1.5 mA) (Schneider et al., 2021; Zhou et al., 2021; Orcioli-Silva et al., 2021). No studies employed intensities above 2.5 mA, which is consistent with the safety threshold for tDCS. Regarding stimulation duration, only one study set the intervention duration to 30 min (Kalkhoran and Khanmohammadi, 2025), while the remaining studies used 20 min.

**Table 2:** tDCS stimulation parameters.

Study	Main electrode	Electrode size (cm^2^)	Intensity (mA)	Duration (min)	Number of sessions	Brain region of stimulation	Position of anode	Position of cathode
Zhou et al. (2021)	Anode	M = R = 3.14	1.5	20	1	L-DLPFC; SM1; L-DLPFC+SM1	F3; Cz; F3, Cz	AF4, CP1, FC1, Cz, FC5 AF4, CP1, FC1, F3, FC5 AF4, CP1, FC1, FC5
Schneider et al. (2021)	Anode	M = R = 3.14	0.5	20	1	L-DLPFC+M1	F3, Cz	AF4, CP1, FC1, FC5
Rodrigues et al. (2023)	Anode	M = R = 25	2	20	16	L-DLPFC	Over the L-DLPFC	Over the supraorbital region
Orcioli-Silva et al. (2021)	Anode	M = 9, R = 25	0.6	20	1	L-PFC + M1	Between AF3 to Fp1, Cz	Right mastoid process
Manor et al. (2018)	Anode	M = R = 35	2	20	10	L-DLPFC	F3	Right supra-orbital margin (Fp2)
Liao et al. (2021)	Anode	M = R = 35	2	20	36	L-DLPFC	F3	Right supraorbital area
Lau et al. (2024)	Anode	M = R = 35	2	20	15	L-DLPFC	F3	Contralateral right supraorbital region(Fp4)
Khanmohammadi et al. (2025a)	Anode	M = R = 3.14	2	20	1	L-DLPFC; M1; Cerebellum	F3; Cz; Iz;	Fz, C3, FP1, F7 Fz, C3, C4, Pz Oz, O2, P8, PO8
Jor'dan et al. (2022)	Anode	M = R = 35	2	20	10	L-DLPFC	F3	Right supra-orbital margin(Fp2)
Baharlouei et al. (2020)	Anode	M = 27, R = 36	2	20	1	Cerebellum; M1	1 cm below the inion of the occipital bone; 1 cm behind the vertex	Over the right shoulder; on the forehead
Kalkhoran and Khanmohammadi (2025)	Anode	NR	2	30	16	M1 + PPC	C3, P4	Fp1, Fp2
Mishra and Thrasher (2022)	Anode	M = R = 35	2	20	1	L-DLPFC; M1	F3; Cz	Over the right supraorbital; the right orbital frontal cortex(Fp2)
Yun et al. (2025)	Anode	M1/DLPFC/Sham-M = 28.27,vmPFC-R = ss6.25	2	20	1	M1; L-DLPFC; vmPFC	Cz; F3; Fpz	The right orbital frontal cortex(Fp2); on the right DLPFC (F4)
Swank et al. (2016)	Anode	NR	2	20	1	DLPFC	L-DLPFC	R-DLPFC
Schabrun et al. (2016)	Anode	M = R = 35	2	20	9	M1	Over the left primary motor cortex	Over the contralateral supra-orbital region

M, main electrode; R, reference electrode; DLPFC, dorsolateral prefrontal cortex; SM1, primary somatosensory cortex; PPC, posterior parietal cortex; vmPFC,ventromedial prefrontal cortex.

In terms of the type of effects, eigh of the 15 studies focused on immediate effects (Schneider et al., 2021; Zhou et al., 2021; Jor'dan et al., 2022; Orcioli-Silva et al., 2021; Baharlouei et al., 2020; Khanmohammadi et al., 2025b; Swank et al., 2016; Yun et al., 2025), primarily assessing short-term neuromodulatory effects through assessments performed immediately after a single session of stimulation or through multiple single sessions within a cross-over design; none of these studies included long-term follow-up. Six studies were long-term effect studies (Lau et al., 2024; Liao et al., 2021; Manor et al., 2018; Kalkhoran and Khanmohammadi, 2025; Schabrun et al., 2016; Rodrigues et al., 2023), with intervention durations of ≥2 weeks (range: 3–12 weeks). Among these, two studies (Manor et al., 2018; Schabrun et al., 2016) included explicit follow-up assessments (at 2 weeks and 12 weeks post-intervention, respectively) to verify the sustainability of the effects, while the remaining four studies only assessed outcomes immediately after the intervention. One study (Mishra and Thrasher, 2022) exhibited characteristics of both immediate and short-term sustained effects, tracking the effects of the intervention 30 min post-stimulation but did not include long-term follow-up. Regarding the application of combined interventions, six studies adopted a protocol in which tDCS was delivered concurrently with other interventions (Schneider et al., 2021; Lau et al., 2024; Liao et al., 2021; Kalkhoran and Khanmohammadi, 2025; Schabrun et al., 2016; Orcioli-Silva et al., 2021). The combined interventions included virtual reality walking in complex environments, treadmill walking, Tai Chi training, interactive computer-based cognitive training, aquatic therapy, and DT gait training. The remaining nine studies involved tDCS alone (Zhou et al., 2021; Manor et al., 2018; Jor'dan et al., 2022; Rodrigues et al., 2023; Baharlouei et al., 2020; Khanmohammadi et al., 2025b; Mishra and Thrasher, 2022; Swank et al., 2016; Yun et al., 2025), with concurrent gait testing or cognitive tasks serving only as outcome assessment tools rather than constituting a combined intervention paradigm.

### 3.5 Outcome measures

All studies employed a DT paradigm, in which participants performed a cognitive task concurrently with a physical task, to evaluate the effects of tDCS on DTP. Ten studies specifically configured the DT paradigm as walking combined with cognitive tasks, including serial subtraction (SST), verbal fluency, and counting (Schneider et al., 2021; Zhou et al., 2021; Lau et al., 2024; Liao et al., 2021; Manor et al., 2018; Jor'dan et al., 2022; Kalkhoran and Khanmohammadi, 2025; Schabrun et al., 2016; Orcioli-Silva et al., 2021; Khanmohammadi et al., 2025b). In these studies, participants’ DTP was primarily measured by DTC and spatiotemporal gait parameters under DT conditions. Gait speed was the most commonly reported outcome, measured in eight studies (Schneider et al., 2021; Zhou et al., 2021; Lau et al., 2024; Liao et al., 2021; Manor et al., 2018; Kalkhoran and Khanmohammadi, 2025; Schabrun et al., 2016; Khanmohammadi et al., 2025b). Gait stability metrics, including stride time variability (STV) and stride length variability (SLV), were included in five studies (Schneider et al., 2021; Zhou et al., 2021; Lau et al., 2024; Manor et al., 2018; Khanmohammadi et al., 2025b). six studies assessed the effects of tDCS on other spatiotemporal gait parameters under DT conditions, such as stride length, cadence, and step length (Schneider et al., 2021; Lau et al., 2024; Liao et al., 2021; Manor et al., 2018; Schabrun et al., 2016; Khanmohammadi et al., 2025b). three of the 10 studies employed a DT paradigm in which participants performed a cognitive task while standing (Schneider et al., 2021; Zhou et al., 2021; Manor et al., 2018), and assessed changes in postural sway area and postural sway speed following tDCS (Zhou et al., 2021; Manor et al., 2018). Four studies used the TUG (Timed Up-and-Go) test as the motor task within the motor-cognitive DT paradigm, with completion time under DT conditions serving as the primary outcome measure (Rodrigues et al., 2023; Mishra and Thrasher, 2022; Swank et al., 2016; Yun et al., 2025). Only one study used changes in center of pressure during a standing DT paradigm as an outcome measure to assess DTP (Baharlouei et al., 2020). In addition to the motor-cognitive DT paradigm, four studies also assessed a motor-motor DT paradigm (Liao et al., 2021; Rodrigues et al., 2023; Swank et al., 2016; Yun et al., 2025), in which participants were instructed to carry a cup of water while walking, standing, or performing the TUG, in order to explore the effects of different types of secondary tasks on DTP in older adults. Moreover, 10 of the included studies further calculated the DTC or DT effect for gait speed, STV, SLV, and TUG time (Schneider et al., 2021; Zhou et al., 2021; Lau et al., 2024; Liao et al., 2021; Manor et al., 2018; Jor'dan et al., 2022; Khanmohammadi et al., 2025b; Mishra and Thrasher, 2022; Swank et al., 2016; Yun et al., 2025), which served as key indicators to quantify the extent of motor-cognitive interference. A summary of the outcome measures used across studies is presented in Table 3.

**Table 3:** Interventions and outcomes.

Study	Intervention	Dual-task paradigm	Outcome measure(s)	Measurement time	Results	Blinding design
Zhou et al. (2021)	tDCS	Standing + SST Walking + SST	DTC、postural sway speed/area, gait speed, STV.	Immediately before and after each tDCS intervention.	L-DLPFC (alone/combined): DTC of postural sway (speed/area) and gait speed↓, STV DTC → .	Double-blind (*p* = 0.29)
Schneider et al. (2021)	tDCS + VR walking, tDCS + Seated rest, sham + VR walking.	Standing + SST Walking + SST	DTC to gait speed、stride time、double support time、STV、swing time variability、step regularity.	Immediately before and after each tDCS intervention.	tDCS + VR walking: Gait DTC ↓, Stroop performance↑,tDCS + seated and sham:→	Double-blind (*p* > 0.29)
Rodrigues et al. (2023)	tDCS	TUG + holding water; TUG + counting odd numbers	TUG time.	At baseline and 8 weeks post-intervention.	TUG performance→.	Single-blind.
Orcioli-Silva et al. (2021)	tDCS+ Mixed treadmill walking	Treadmill walking + Digit vigilance task	Cadence,stance time ratio, STV, SLV.	Immediately before and after the intervention.	Cadence、stance time ratio、STV、SLV → .	Double blind.
Manor et al. (2018)	tDCS	Standing + SST Walking + SST	DTC, gait speed,stride time, STV, postural sway speed, postural sway area.	Baseline, before and after the first intervention, post-intervention, 2 weeks post-intervention.	DTC↓, DT- stride time↓, DT-STV↓.	Double blind (*p* = 0.76)
Liao et al. (2021)	tDCS + Yang Style Tai Chi	Walking + carrying a water tray; Walking + SST	DTC, gait speed, cadence, stride length.	Baseline, post-12-week intervention, 12-week follow-up.	DTC↓, DT- stride length↑, DT- step time variability↓, ST- spatiotemporal parameter→.	Double blind.
Lau et al. (2024)	tDCS + ICCT	Walking + SST	DTC, gait speed, cadence, stride length, gait variability.	Baseline, post-intervention (1 day post-15 sessions).	DTC↓, DT-spatiotemporal gait parameters→.	Double blind.
Khanmohammadi et al. (2025a)	HD-tDCS	Walking + SST	DTC, gait speed, stride time, STV, stride length, SLV.	Pre-tDCS (baseline), immediately post-tDCS.	M1, LDLPFC: DT- gait speed↑, other outcome measures→.	Double blind: effective (binomial test, *p* < 0.001)
Jor'dan et al. (2022)	tDCS	Walking + SST/Walking+ verbal fluency	DTC, gait speed.	Pre-stimulation, immediately post-stimulation.	DTC↓, gait speed→.	Double blind.
Baharlouei et al. (2020)	tDCS	Standing + SST	CoP indices in ML/AP/resultant directions.	Immediately before and after the intervention.	TPL/MV (ML/AP) in DT↓, CoPD→.	Double blind:(*p* > 0.05)
Kalkhoran and Khanmohammadi (2025)	tDCS,tDCS+ aquatic therapy	Walking + visuospatial N-back task	Gait speed in DT.	Baseline (pre-intervention) and post 8-week intervention.	Gait speed in DT↑.	Triple-blind.
Mishra and Thrasher (2022)	tDCS	iTUG + phoneme verbal fluency task	DTC,iTUG time.	baseline (before), during (15 min after stimulation start), immediately after, 15 min post, 30 min post stimulation	correct words/min↑, DTC→, iTUG time→.	Double blind.
Yun et al. (2025)	tDCS	TUG + SST; TUG+carrying water cup	TUG time, DTE.	Immediately before and after each stimulation session	M1 tDCS: TUG time↓; All groups: DTE → .	Double blind.
Swank et al. (2016)	tDCS	TUG+SST; TUG+carrying full water cup.	DTC, TUG time.	All outcomes measured after each tDCS session; baseline assessments conducted before intervention initiation.	DTC↓, TUG time→.	Double blind.
Schabrun et al. (2016)	Dual task gait training + tDCS	Walking+ cognitive tasks (word lists/counting/conversation), TUG+SST/word list generation.	Gait parameters (speed/cadence/step length/double support time), TUG time.	All outcomes measured pre-intervention (baseline), post-3-week intervention, and 12-week follow-up.	Dual-task cognitive performance↑.	Double blind.

SST, serial subtraction task; VR, virtual reality; ICCT,interactive computerized cognitive training;iTUG, interactive computerized cognitive training; STV, stride time variability; SLV, stride length variability; NR, not reported. ↑, indicates an increase in the index value; ↓, indicates a decrease in the index value; →, indicates no significant change in the value.

### 3.6 Blinding efficacy

As shown in Table 3, 13 of the 15 studies were double-blind (Schneider et al., 2021; Zhou et al., 2021; Lau et al., 2024; Liao et al., 2021; Manor et al., 2018; Jor'dan et al., 2022; Schabrun et al., 2016; Orcioli-Silva et al., 2021; Baharlouei et al., 2020; Khanmohammadi et al., 2025b; Mishra and Thrasher, 2022; Swank et al., 2016; Yun et al., 2025). One study used a triple-blind design, in which participants, therapists, and outcome assessors were all blinded (Kalkhoran and Khanmohammadi, 2025). Only one study employed a single-blind design, with blinding applied solely to the participants (Rodrigues et al., 2023). Among all studies, only five reported blinding efficacy and explicitly confirmed the effectiveness of blinding implementation (Schneider et al., 2021; Zhou et al., 2021; Manor et al., 2018; Khanmohammadi et al., 2025b; Mishra and Thrasher, 2022).

### 3.7 Side effects of tDCS

Five of the 15 studies did not report whether adverse events occurred (Jor'dan et al., 2022; Kalkhoran and Khanmohammadi, 2025; Rodrigues et al., 2023; Khanmohammadi et al., 2025b; Swank et al., 2016). Among studies that provided safety data, reported events were generally mild and transient, including skin redness, tingling, headache, neck pain, and drowsiness, and no serious adverse events were reported. However, incomplete adverse-event reporting, small samples, and short follow-up preclude firm conclusions regarding safety.

### 3.8 Effects of tDCS on DTP in older adults

Thirteen of the 15 studies reported improvement in at least one gait, mobility, balance, or DTC outcome under DT conditions, whereas two reported no significant motor improvement (Orcioli-Silva et al., 2021; Rodrigues et al., 2023). Because many studies assessed multiple outcomes and concurrent cognitive-task performance was inconsistently reported, these study-level findings should not be interpreted as evidence that overall DTP improved. Evidence of persistence beyond the immediate post-intervention period was limited to a small number of studies (Liao et al., 2021; Manor et al., 2018; Schabrun et al., 2016).

### 3.9 Meta-analysis

Among the 15 included studies, only one used the center of pressure to assess the effects of tDCS on DTP in older adults (Baharlouei et al., 2020). Two other studies did not report the mean or standard deviation values for DT outcomes, and the authors did not respond to our requests for additional data (Jor'dan et al., 2022; Mishra and Thrasher, 2022). Therefore, these three studies were excluded from the meta-analysis. The final quantitative synthesis included the remaining 12 studies. The primary meta-analysis included only healthy or community-dwelling older adults. Due to the small number of studies available for these prespecified analyses, separate forest plots were not generated; pooled effect sizes, 95% CIs, *p*-values and heterogeneity statistics are presented in Table 4. Figures 3–10 show exploratory pooled estimates combining both healthy and clinical participants. These mixed-population results are hypothesis-generating only and should not be interpreted as equivalent to the prespecified primary or secondary analyses. Supplementary Tables S1, S2 report the crossover-correlation sensitivity analyses; Supplementary Tables S3, S4 provide the GRADE and evidence-summary tables; and Supplementary Table S5 lists every comparison-level effect before within-study consolidation.

**Table 4:** Summary of meta-analysis results.

Outcome measure	Populations	*N*	Heterogeneity		Hedges’ *g* (95% CI)	Meta analysis	
			*I*^2^ (%)	*p*		*z*	*p*
Primary meta-analysis: healthy populations							
Gait speed	Healthy primary	2	0.0	0.354	0.200 (0.017 to 0.383)	2.144	0.032
DTC of gait speed	Healthy primary	2	0.0	0.795	−0.368 (−0.557 to −0.179)	−3.814	<0.001
DTC of stride time	Healthy primary	2	84.0	0.013	0.032 (−0.610 to 0.675)	0.099	0.921
STV	Healthy primary	2	63.6	0.097	−0.181 (−0.525 to 0.164)	−1.026	0.305
DTC of STV	Healthy primary	4	94.5	<0.001	0.515 (−0.441 to 1.471)	1.056	0.291
DTC of SLV	Healthy primary	2	79.3	0.028	−0.015 (−1.087 to 1.057)	−0.028	0.978
Secondary meta-analysis: clinical populations							
Gait speed	Clinical secondary	4	31.1	0.225	−0.158 (−0.607 to 0.290)	−0.691	0.490
DTC of gait speed	Clinical secondary	3	0.0	0.409	−0.319 (−0.815 to 0.177)	−1.259	0.208
Cadence	Clinical secondary	3	63.0	0.067	0.332 (−0.457 to 1.121)	0.824	0.410
Stride length	Clinical secondary	2	0.0	0.377	0.328 (−0.267 to 0.922)	1.080	0.280
TUG time	Parkinson disease secondary	2	30.7	0.230	−0.241 (−0.641 to 0.159)	−1.180	0.238
Not pooled within population	Stride time; healthy stride length/TUG; sway outcomes	1per population	—	—	Narrative synthesis	—	—

Hedges’ *g*: represents the standardized mean difference, with 95% confidence intervals (CI). Positive Hedges’ *g* values denote improved performance following tDCS intervention, whereas negative values indicate deteriorated performance. *I*^2^ and *p*-values describe between-study heterogeneity. *Z*-statistic and corresponding p-values reflect the overall effect size from pooled meta-analysis. Primary meta-analysis included healthy participants; secondary meta-analysis included clinical populations. Outcomes with only one available study per population were not quantitatively pooled and were summarized via narrative synthesis. DTC, dual-task cost; STV, stride-time variability; SLV, stride-length variability; TUG, Timed Up-and-Go test.

**Figure 3:**
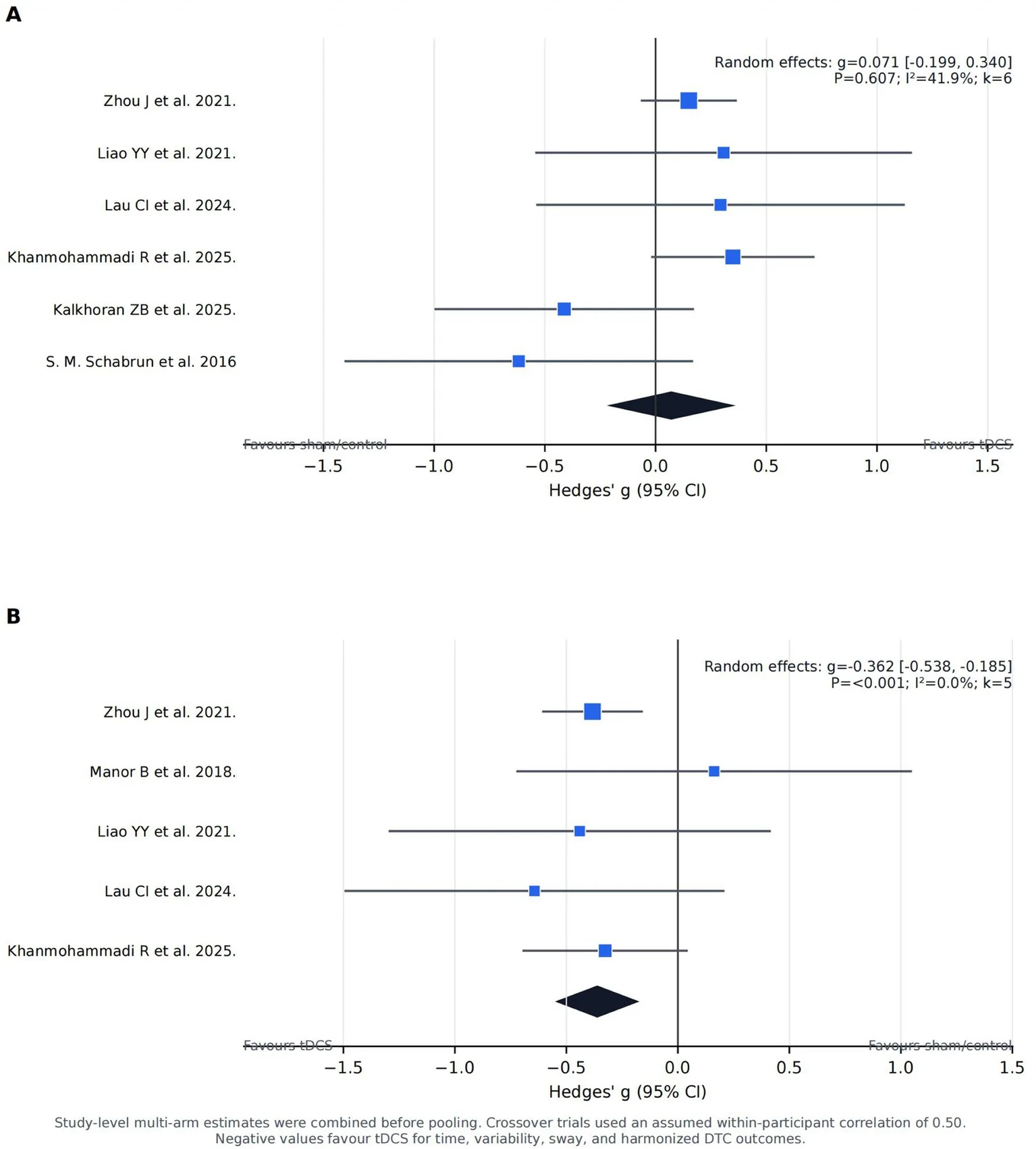
Study-level forest plots for **(A)** gait speed and **(B)** DTC of gait speed. Printed pooled estimates combine all eligible studies and are exploratory when healthy and clinical populations are mixed; the prespecified healthy-only and clinical-only estimates are reported separately in Table 4 and the Results.

**Figure 4:**
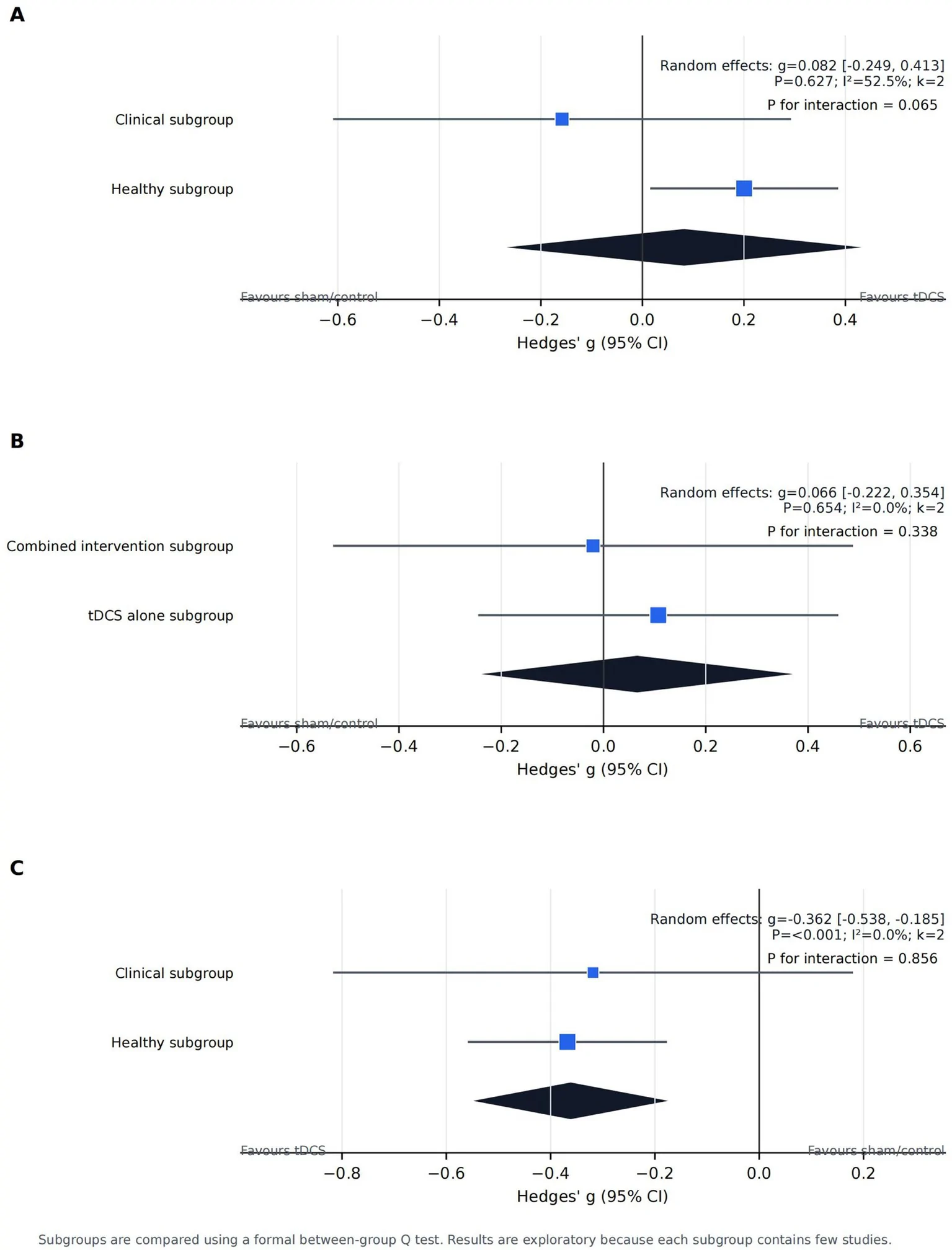
Exploratory population and intervention comparisons. These analyses are secondary and do not replace the healthy-older-adult primary meta-analysis. Differences are interpreted only from formal interaction tests. **(A)** Gait speed by population. **(B)** Gait speed by intervention type. **(C)** Gait speed DTC by population.

**Figure 5:**
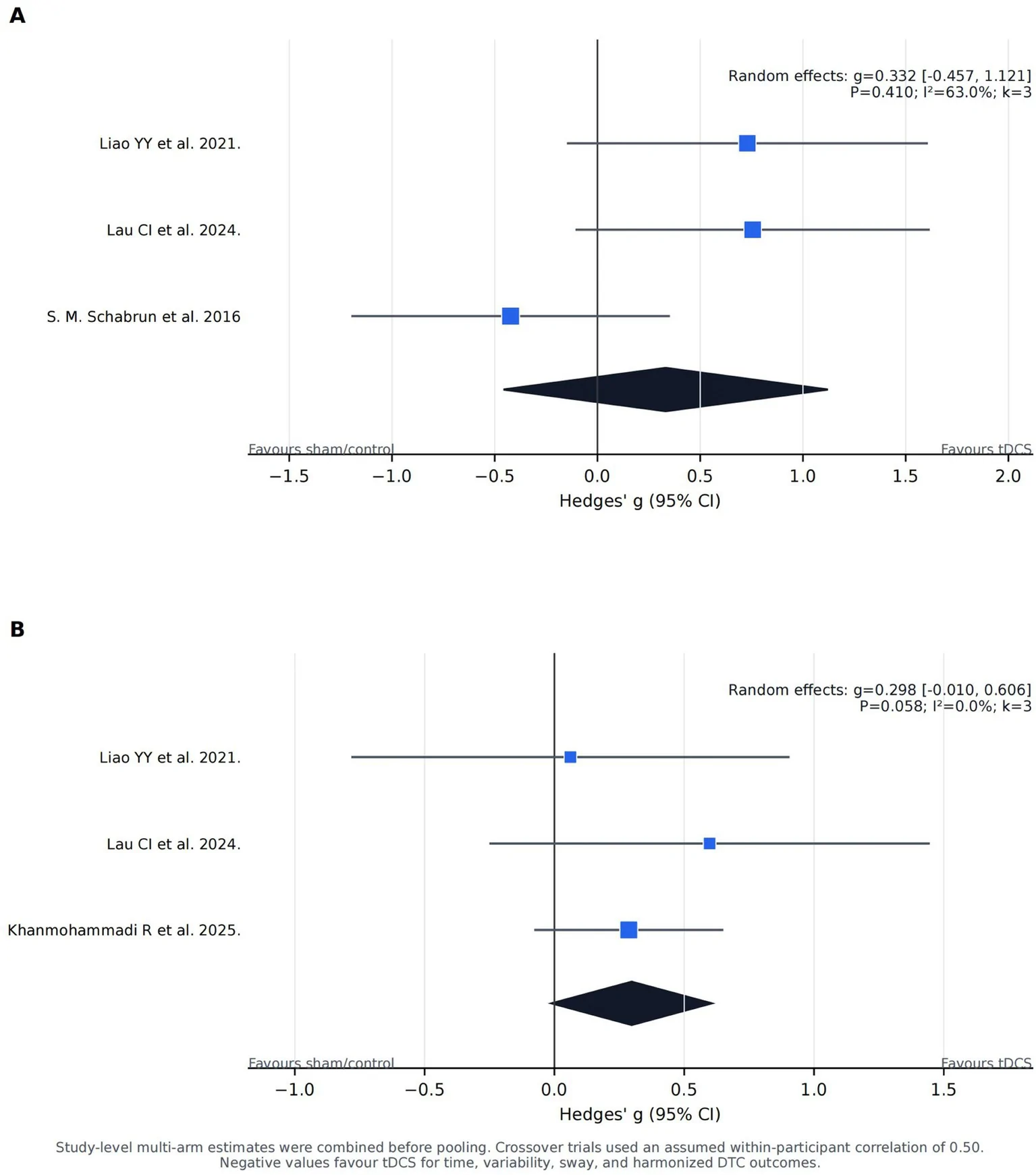
Study-level forest plots for **(A)** cadence and **(B)** stride length. Cadence includes clinical studies only; stride length combines healthy and clinical studies and is exploratory. Corresponding population-specific estimates are reported separately in Table 4.

**Figure 6:**
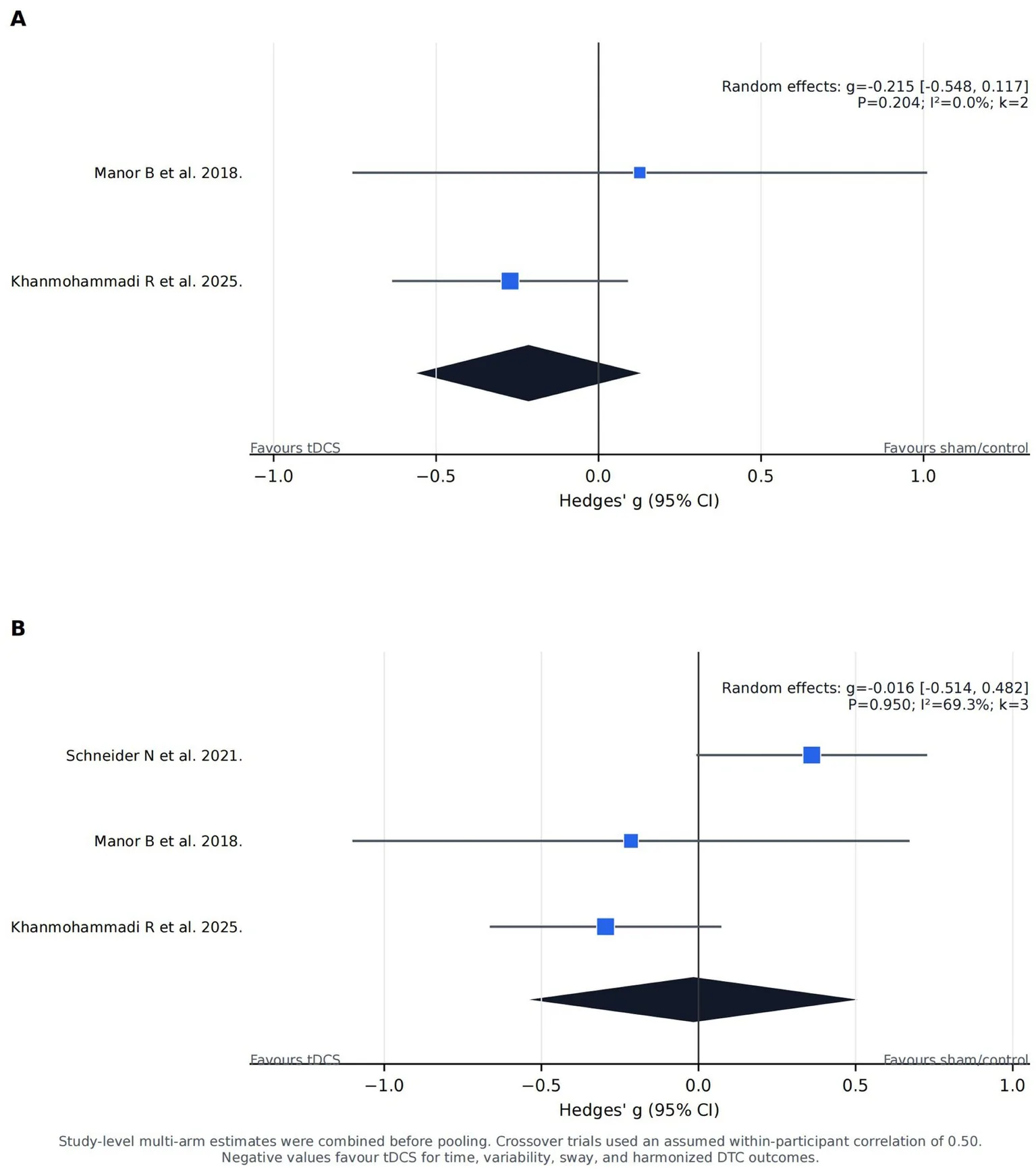
Study-level forest plots for **(A)** stride time and **(B)** DTC of stride time. Printed pooled estimates combine all eligible studies; the healthy-only DTC analysis is reported separately in Table 4 and the Results.

**Figure 7:**
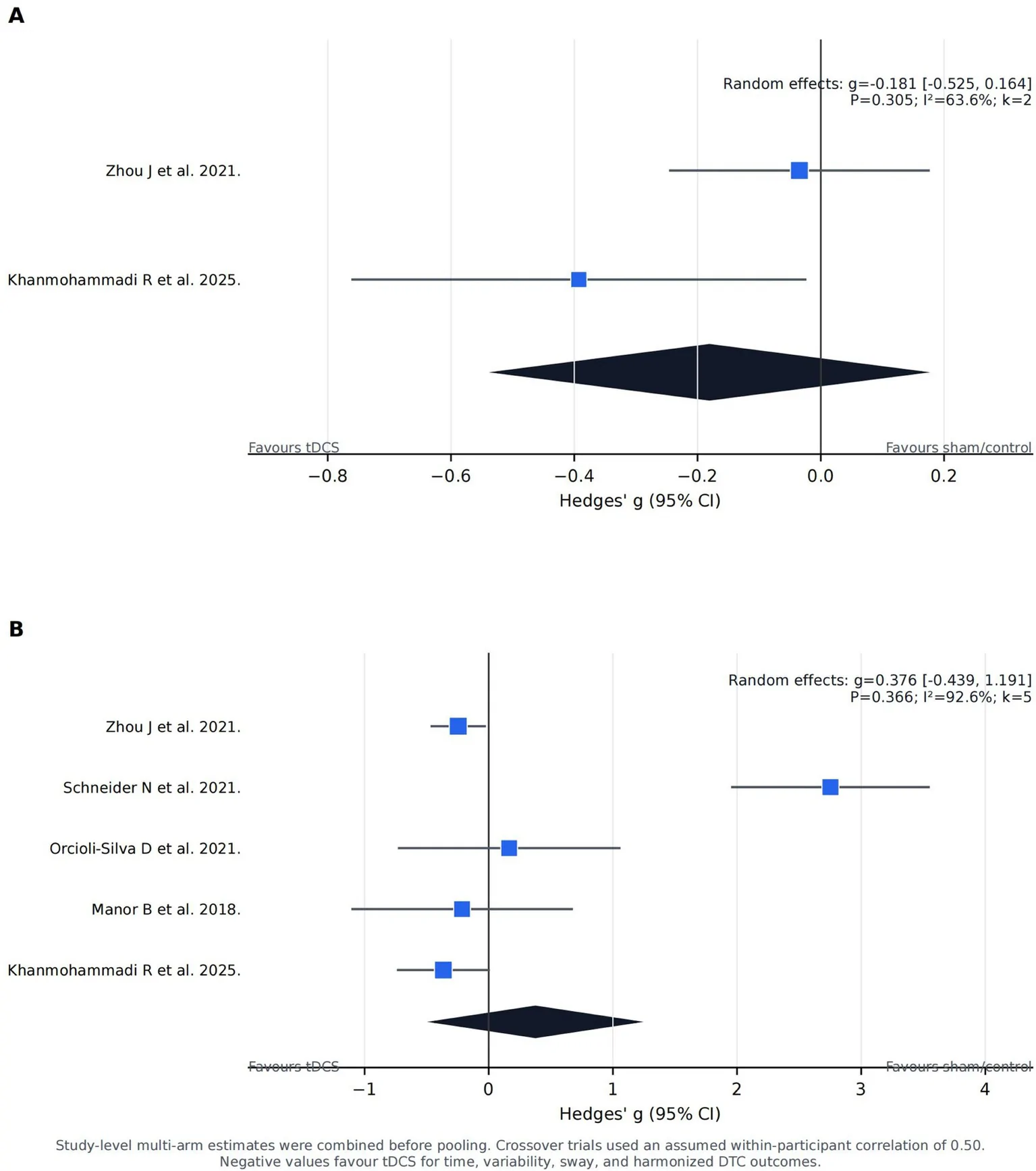
Study-level forest plots for **(A)** STV and **(B)** DTC of STV. The STV forest plot contains healthy studies only; the DTC-of-STV forest plot combines healthy and clinical populations and is exploratory. The healthy-only estimate is reported separately in Table 4.

**Figure 8:**
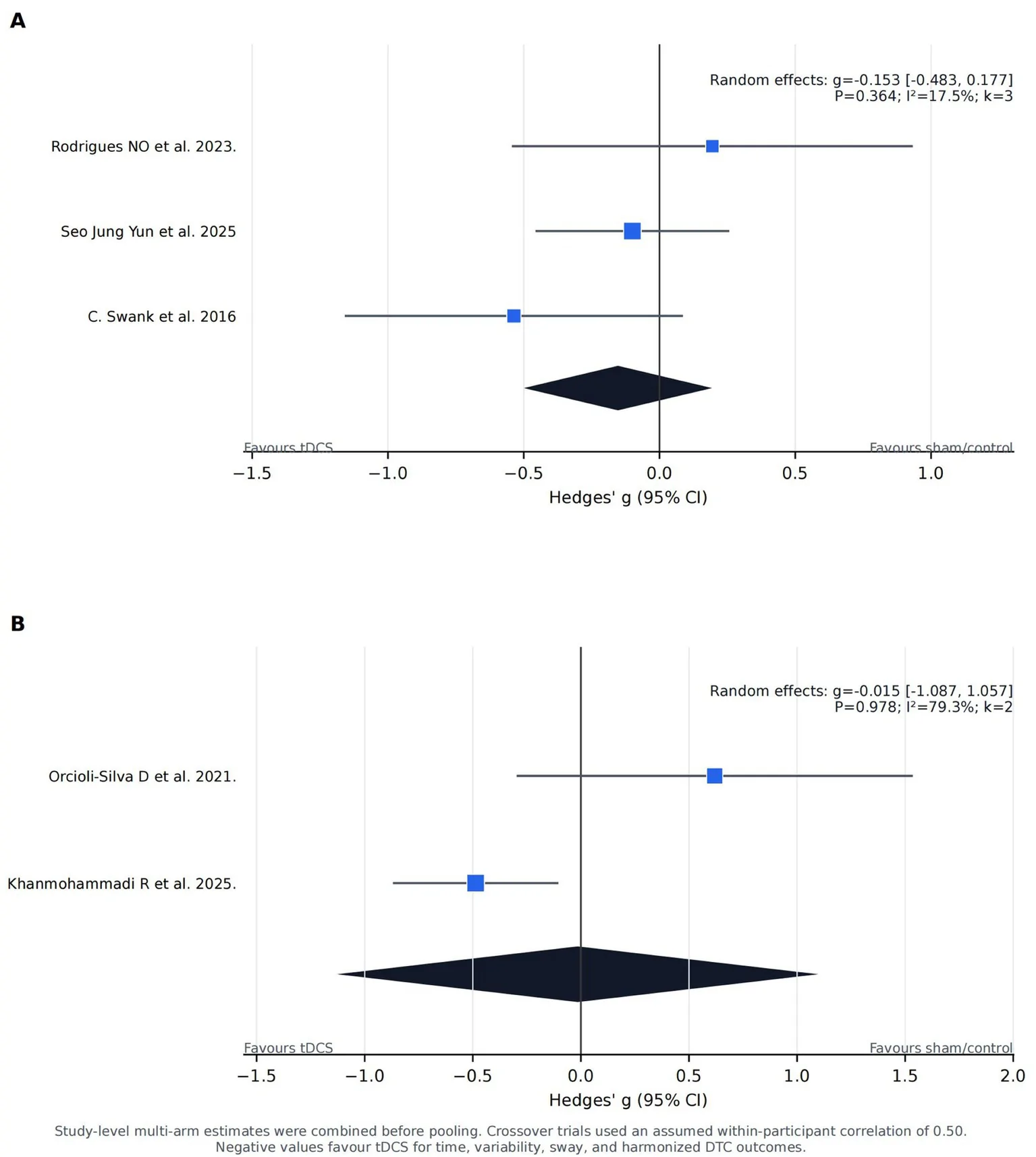
Study-level forest plots for **(A)** TUG time and **(B)** DTC of SLV. TUG combines healthy and clinical studies and is exploratory; DTC of SLV contains healthy studies only and corresponds to the primary healthy-population estimate.

**Figure 9:**
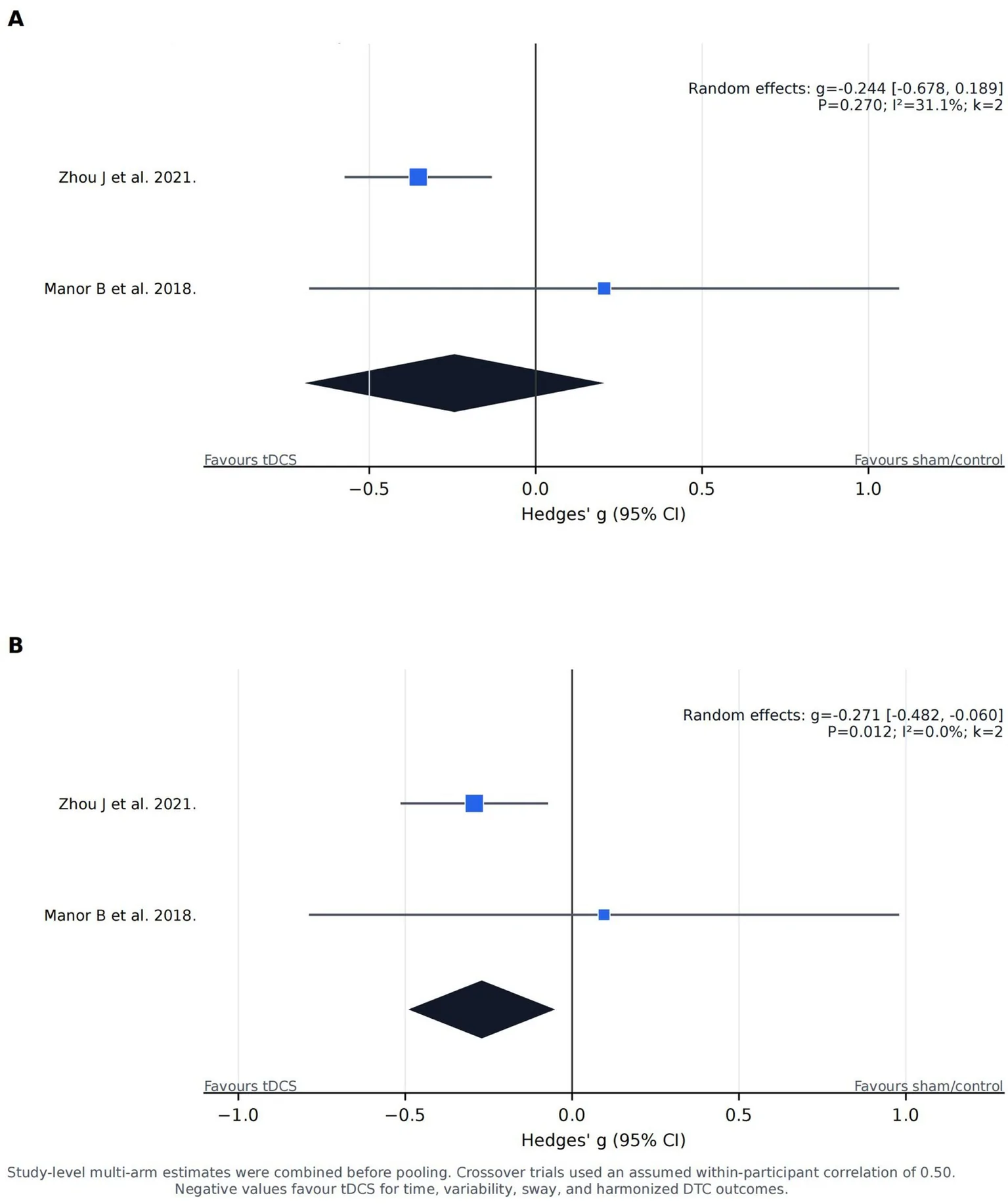
Study-level forest plots for **(A)** standing sway speed and **(B)** its DTC. Each pooled estimate combines one healthy and one clinical study and must be interpreted as exploratory.

**Figure 10:**
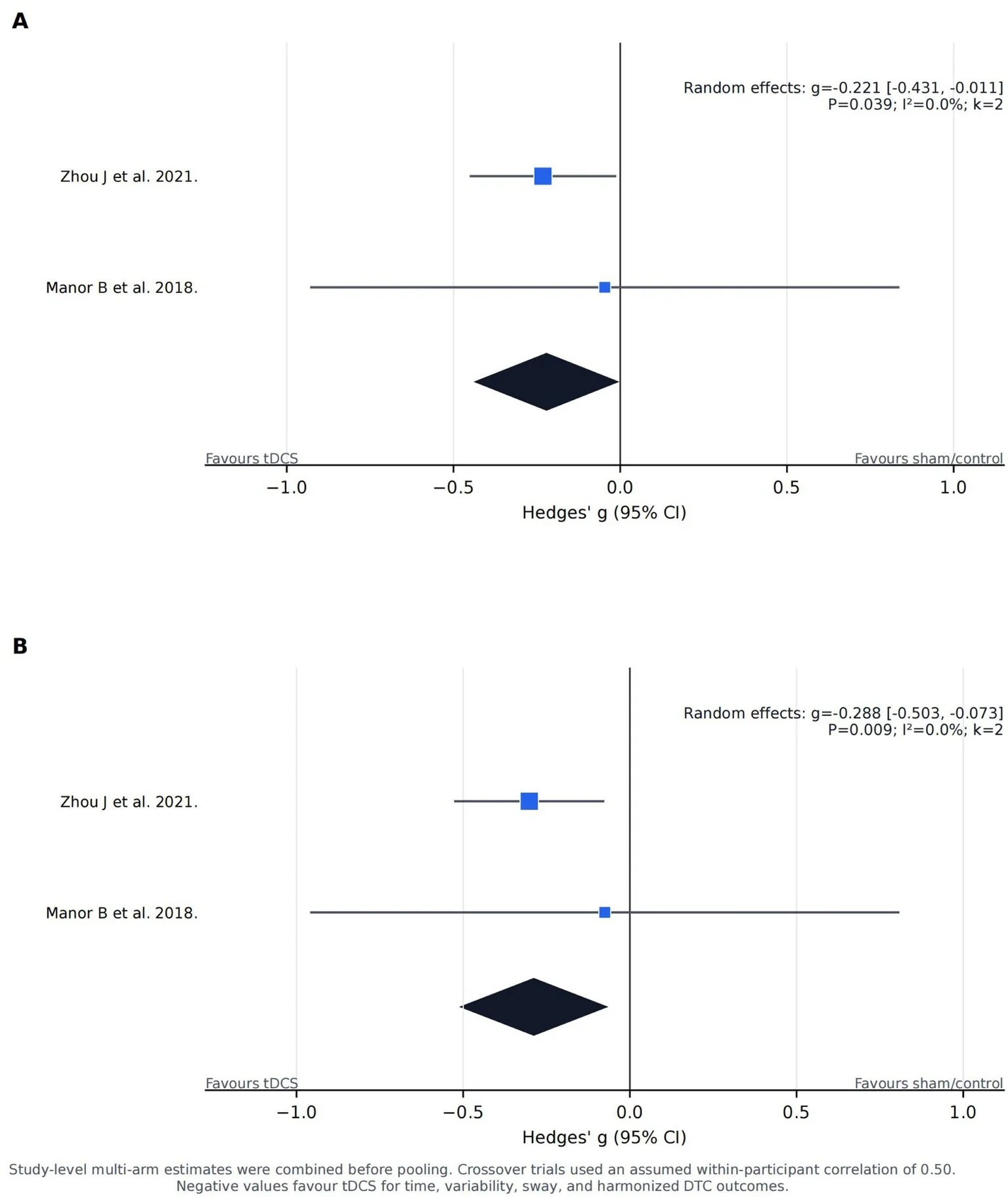
Study-level forest plots for **(A)** standing sway area and **(B)** its DTC. Each pooled estimate combines one healthy and one clinical study and must be interpreted as exploratory.

#### 3.9.1 Gait speed

Figure 3A shows that the exploratory all-study analysis of gait speed did not demonstrate a significant effect of tDCS (Hedges’ *g* = 0.071, 95% CI -0.199 to 0.340; *p* = 0.607; *I*^2^ = 41.9%). Exploratory subgroup comparisons for gait speed and its dual-task cost are presented in Table 5. In contrast, the prespecified analysis restricted to healthy older adults (Zhou et al., 2021; Khanmohammadi et al., 2025b) yielded a small positive effect (Hedges’ *g* = 0.200, 95% CI 0.017 to 0.383; *p* = 0.032; *I*^2^ = 0%). Four clinical-population studies (Lau et al., 2024; Liao et al., 2021; Kalkhoran and Khanmohammadi, 2025; Schabrun et al., 2016) yielded no clear effect (Hedges’ *g* = −0.158, 95% CI − 0.607 to 0.290; *p* = 0.490; *I*^2^ = 31.1%). Because diagnoses and co-interventions differed, the clinical estimate is secondary and should not be generalized across conditions. The apparent discrepancy between the figure-level and primary estimates reflects the different populations included rather than a calculation error.

**Table 5:** Subgroup analysis of dual-task gait speed and DTC.

Subgruop	*N*	Heterogeneity		Hedges’ *g* (95% CI)	Meta analysis		*p* for subgroup difference
		*I*^2^ (%)	*p*		*z*	*p*	
Exploratory subgroup comparison for gait speed (not the primary analysis)							
Population type							
Healthy	2	0.0	0.354	0.200 (0.017 to 0.383)	2.147	0.032	**0.065**
Impaired	4	31.1	0.225	−0.158(−0.607 to 0.291)	−0.691	0.490	
Intervention type							
Alone	3	58.7	0.089	0.107 (−0.243 to 0.457)	0.600	0.549	**0.338**
Combined	3	29.6	0.242	−0.021 (−0.527 to 0.486)	−0.079	0.937	
Exploratory subgroup comparison for DTC of gait speed (not the primary analysis)							
Population type							
Healthy	2	0.0	0.795	−0.368(−0.557 to −0.179)	−3.817	<0.001	**0.856**
Impaired	3	0.0	0.409	−0.319 (−0.815 to 0.178)	−1.258	0.208	

Hedges’*g* is the standardized mean difference with 95% confidence intervals (CI). *I*^2^ and *p*-values represent study heterogeneity. *Z*-statistic and *p*-values indicate pooled overall effects. *p* for subgroup difference tests between-subgroup heterogeneity. These are exploratory analyses and not predefined primary outcomes. DTC, dual-task cost.

For the DTC of gait speed, Figure 3B shows a significant reduction favouring tDCS in the exploratory all-study analysis (Hedges’ *g* = −0.362, 95% CI -0.538 to −0.185; *p* < 0.001; *I*^2^ = 0.0%). Two healthy-older-adult studies (Zhou et al., 2021; Khanmohammadi et al., 2025b) showed a reduction at corr = 0.50 (Hedges’ *g* = −0.368, 95% CI − 0.557 to −0.179; *p* < 0.001; *I*^2^ = 0%). Three clinical-population studies (Lau et al., 2024; Liao et al., 2021; Manor et al., 2018) were inconclusive (*g* = −0.319, 95% CI − 0.815 to 0.177; *p* = 0.208; *I*^2^ = 0%). The healthy estimate remained significant across corr assumptions, although it was based on only two studies.

#### 3.9.2 Cadence and stride length

As shown in Figure 5A, three clinical-population studies^(34, 35, 39)^produced an inconclusive pooled estimate for cadence (Hedges’ *g* = 0.332, 95% CI − 0.457 to 1.121; *p* = 0.410; *I*^2^ = 63.0%). Because no eligible healthy-older-adult study contributed to this outcome, this estimate represents a secondary clinical analysis rather than a primary healthy-population effect.

Figure 5B shows that the exploratory mixed-population estimate for stride length did not reach statistical significance (Hedges’ *g* = 0.298, 95% CI -0.010 to 0.606; *p* = 0.058; *I*^2^ = 0.0%). Only one healthy study (Khanmohammadi et al., 2025b) reported stride length, precluding a primary meta-analysis. Two clinical studies (Lau et al., 2024; Liao et al., 2021) yielded an inconclusive secondary estimate (Hedges’ *g* = 0.328, 95% CI − 0.267 to 0.922; *p* = 0.280; *I*^2^ = 0%). The exploratory mixed-population estimate was sensitive to corr and is reported in Supplementary Table S2.

#### 3.9.3 Stride time

The exploratory forest plot in Figure 6A included one healthy study (Khanmohammadi et al., 2025b) and one clinical study^(36)^and showed no statistically significant effect on stride time (Hedges’ *g* = −0.215, 95% CI -0.548 to 0.117; *p* = 0.204; *I*^2^ = 0.0%). Because each population contributed only one study, neither a healthy-primary nor a clinical-secondary pooled estimate could be calculated.

Figure 6B shows no significant effect on DTC of stride time in the exploratory all-study analysis (Hedges’ *g* = −0.016, 95% CI -0.514 to 0.482; *p* = 0.950; *I*^2^ = 69.3%). Two healthy studies (Schneider et al., 2021; Khanmohammadi et al., 2025b) yielded no clear effect on DTC of stride time (Hedges’ *g* = 0.032, 95% CI − 0.610 to 0.675; *p* = 0.921; *I*^2^ = 84.0%). The high heterogeneity indicates that stimulation context and DTC definition may have differed materially between studies.

#### 3.9.4 Stride time variability

For STV, Figure 7A included two healthy studies (Zhou et al., 2021; Khanmohammadi et al., 2025b) and therefore coincided with the primary analysis (Hedges’ *g* = −0.181, 95% CI − 0.525 to 0.164; *p* = 0.305; *I*^2^ = 63.6%). Figure 7B shows that the exploratory estimate for DTC of STV was also inconclusive and highly heterogeneous (Hedges’ *g* = 0.376, 95% CI −0.439 to 1.191; *p* = 0.366; *I*^2^ = 92.6%; *k* = 5). Restricting the analysis to four healthy studies (Schneider et al., 2021; Zhou et al., 2021; Orcioli-Silva et al., 2021; Khanmohammadi et al., 2025b) likewise yielded no significant effect (Hedges’ *g* = 0.515, 95% CI − 0.441 to 1.471; *p* = 0.291; *I*^2^ = 94.5%).

The variability results were not materially altered by corr = 0.25 or 0.75: the direction remained similar, all CIs crossed the null, and heterogeneity remained moderate to very high. This supports an interpretation of uncertainty rather than benefit.

#### 3.9.5 Stride length variability

Figure 8B shows no significant effect on DTC of SLV (Orcioli-Silva et al., 2021; Khanmohammadi et al., 2025b) (Hedges’ *g* = −0.015, 95% CI − 1.087 to 1.057; *p* = 0.978; *I*^2^ = 79.3%). Both studies enrolled healthy older adults, so the forest-plot estimate is identical to the prespecified healthy-primary analysis. The wide CI and substantial heterogeneity indicate uncertainty.

#### 3.9.6 TUG

The exploratory all-study analysis shown in Figure 8A found no statistically significant effect on TUG time (Hedges’ *g* = −0.153, 95% CI -0.483 to 0.177; *p* = 0.364; *I*^2^ = 17.5%). Only one community-dwelling study (Rodrigues et al., 2023) reported TUG time, so a primary meta-analysis was not performed. Two Parkinson’s disease studies (Mishra and Thrasher, 2022; Swank et al., 2016) yielded an inconclusive clinical estimate (Hedges’ *g* = −0.241, 95% CI − 0.641 to 0.159; *p* = 0.238; *I*^2^ = 30.7%).

#### 3.9.7 Standing balance

For standing balance, Figure 9A showed no significant effect on sway speed (Hedges’ *g* = −0.244, 95% CI -0.678 to 0.189; *p* = 0.270; *I*^2^ = 31.1%), whereas Figure 9B showed a significant reduction in DTC of sway speed (Hedges’ *g* = −0.271, 95% CI -0.482 to −0.060; *p* = 0.012; *I*^2^ = 0.0%). Figure 10A showed a reduction in sway area (Hedges’ *g* = −0.221, 95% CI -0.431 to −0.011; *p* = 0.039; *I*^2^ = 0.0%), and Figure 10B showed a reduction in DTC of sway area (Hedges’ *g* = −0.288, 95% CI -0.503 to −0.073; *p* = 0.009; *I*^2^ = 0.0%). Population-specific pooling was not possible for standing sway speed or area because one healthy study (Zhou et al., 2021) and one functionally limited study (Manor et al., 2018) contributed to each outcome. The same limitation applied to their DTC outcomes. These results were therefore described narratively, and the previous mixed-population syntheses were moved to the exploratory supplementary analysis.

Across the population-specific analyses, the only statistically significant primary estimates at corr = 0.50 were gait speed and DTC of gait speed in healthy older adults. The remaining primary outcomes were inconclusive or could not be pooled. This distinction is reflected consistently in Table 4, the Discussion, and the Conclusion.

### 3.10 Sensitivity analysis

For healthy older adults, the gait-speed estimate remained positive but its statistical significance depended on corr: corr = 0.25, *g* = 0.203 (95% CI − 0.020 to 0.426), *p* = 0.074, *I*^2^ = 0%; corr = 0.50, *g* = 0.200 (0.017 to 0.383), *p* = 0.032, *I*^2^ = 0%; and corr = 0.75, *g* = 0.215 (0.022 to 0.408), *p* = 0.029, *I*^2^ = 42.5%. Thus, the direction was stable but the significance was not robust. In contrast, DTC of gait speed remained significant at corr = 0.25, 0.50, and 0.75 (g from −0.381 to −0.332; all *p* ≤ 0.001; *I*^2^ = 0%). DTC of stride time, STV, DTC of STV, and DTC of SLV remained non-significant across all three corr values. In the exploratory mixed-population analyses, stride length and sway area changed statistical significance across corr assumptions, whereas effect directions were stable. Complete results are provided in Supplementary Tables S1, S2. Leave-one-out sensitivity analyses for STV and its DTC in the exploratory mixed-population analysis are presented in Table 6.

**Table 6:** Sensitivity analysis (leave-one-out) for STV and STV-DTC.

Sensitivity analysis (leave-one-out method) for STV					
Excluded study	Heterogeneity		Hedges’ *g* (95% CI)	Meta analysis	
	*I*^2^ (%)	*p*		*z*	*p*
Khanmohammadi et al. (2025a)	54.031	0.140	−0.284(−0.916–0.347)	−0.882	0.378
Manor et al. (2018)	57.406	0.125	−0.206(−0.521–0.108)	−1.284	0.199
Zhou et al. (2021)	0.000	0.451	−0.456(−0.794 − −0.119)	−2.649	0.008

Sensitivity analysis (leave-one-out method) for DTC of STV					
Excluded study	Heterogeneity		Hedges’ *g* (95% CI)	Meta analysis	
	*I*^2^ (%)	*p*		*z*	*p*
Zhou et al. (2021)	94.0	<0.001	0.574 (−0.838 to 1.987)	0.797	0.425
Schneider et al. (2021)	0.0	0.747	−0.256 (−0.433 to −0.080)	−2.845	0.004
Orcioli-Silva et al. (2021)	94.4	<0.001	0.429 (−0.523 to 1.382)	0.883	0.377
Manor et al. (2018)	94.5	<0.001	0.515 (−0.441 to 1.471)	1.056	0.291
Khanmohammadi et al. (2025a)	94.2	<0.001	0.602 (−0.732 to 1.936)	0.884	0.376

Leave-one-out sensitivity analyses for STV and its DTC in the exploratory mixed-population analysis, showing the impact of individual studies on pooled effect sizes and heterogeneity.

### 3.11 Reporting bias

No outcome included 10 or more studies; therefore, funnel plots and statistical tests of asymmetry were not performed because they would have insufficient power to distinguish publication bias from chance.

### 3.12 Certainty of evidence

Certainty of evidence was assessed separately for the healthy primary analyses and the eligible clinical secondary analyses. The GRADE summary-of-findings tables were moved to Supplementary Tables S3, S4. The overall certainty of evidence for DT gait outcomes in healthy older adults is low or very low. Only gait speed and its DTC, each based on two studies (Zhou et al., 2021; Khanmohammadi et al., 2025b) were rated as moderate certainty. All other variability-related outcomes (including DTC of stride time, STV, DTC of STV, and DTC of SLV) were rated as low or very low certainty. Evidence from clinical populations was also of insufficient certainty, and no reliable pooled effect estimates were obtained.

Moderate or lower certainty indicates that the current evidence provides only limited confidence in the effect of tDCS on DT gait in healthy older adults. Even when meta-analyses show statistically significant results, the true intervention effect may still differ substantially from the current pooled estimates. Moreover, the improvement in gait speed was sensitive to the assumed correlation in crossover trials, undermining its robustness; only the DTC of gait speed remained stable across different correlation assumptions. This evidence profile does not support the use of tDCS as a routine clinical intervention to improve DT gait in healthy older adults. The actual benefits of this intervention await further confirmation from more high-quality, large-sample RCTs.

## 4 Discussion

The RoB 2.0 assessment revealed that reporting bias (D5) was the most common methodological limitation shared by both types of trials, followed by selection bias arising from inadequate randomization procedures. In addition, attrition bias was prominent in the crossover trials. Reporting bias may have inflated the pooled effect estimates, overstating the benefits of tDCS intervention and thereby reducing the credibility of the conclusions. Future RCTs should be prospectively registered and all prespecified outcomes should be fully reported in accordance with the CONSORT statement. Parallel RCTs should improve randomization and allocation concealment procedures. Crossover trials should incorporate adequate washout periods, handle dropout data systematically, and implement blinding of outcome assessment. These measures would enhance the quality of primary research.

This study is the first to systematically and quantitatively assess the effects of tDCS on DTP in older adults, with a primary focus on four dimensions: spatiotemporal gait parameters under DT conditions (e.g., gait speed, cadence, stride time), TUG performance, standing balance, and DTC. At corr = 0.50, gait speed and DTC of gait speed favoured tDCS, whereas DTC of stride time, STV, DTC of STV, and DTC of SLV were inconclusive. Cadence, stride length, stride time, TUG, and standing-balance outcomes could not be pooled in the healthy population because fewer than two comparable studies were available. Clinical-population estimates were analysed separately and were generally inconclusive. Thus, the data do not support a broad claim that tDCS improves dual-task gait and balance across older populations.

As the “sixth vital sign” for assessing the overall health status of older adults (Bortone et al., 2021; Atrsaei et al., 2021), gait speed is not only a simple motor function indicator but is also closely related to fall risk, all-cause mortality, independent living ability, and quality of life in the elderly (Studenski et al., 2011; Luo et al., 2023; Montero-Odasso et al., 2012). Capacity-sharing theory proposes that walking and a concurrent cognitive task draw on a limited pool of executive and attentional resources (Tsang et al., 2022) Older adults often compensate for reduced automaticity by recruiting prefrontal control, leaving less reserve when both tasks are performed together (Clark, 2015; Liu et al., 2024; Wei et al., 2020). Anodal DLPFC stimulation could increase cortical excitability or network efficiency, reduce the amount of prefrontal resource needed for task monitoring, or improve allocation between motor and cognitive goals (Beurskens and Bock, 2012; Manor et al., 2016; Pino-Esteban et al., 2021; Yogev-Seligmann et al., 2008). This mechanism is compatible with the reduced DTC of gait speed in healthy older adults: tDCS may have improved the relative preservation of gait when attention was divided. However, the corr-sensitive gait-speed result and sparse cognitive-task data prevent us from concluding that total dual-task capacity increased; a motor benefit could still reflect a shift in task priority.

The bottleneck model provides a complementary explanation. According to Pashler’s central bottleneck model, sensorimotor tasks involve three serial stages: perceptual, central, and motor. The central stage (e.g., response selection) can process only one task at a time. Thus, when gait adaptation and cognitive response selection require overlapping central operations, processing may become serial rather than parallel (Lee and Han, 2020). tDCS might facilitate operations before or after the bottleneck—such as maintaining task rules, switching attention, preparing a motor response, or monitoring errors—but it is unlikely to remove the structural response-selection bottleneck itself (Zhou et al., 2015; Abedanzadeh et al., 2021). Consequently, an effect may be detectable for a relatively global measure such as gait-speed DTC while remaining absent for outcomes that depend on multiple downstream timing, sensorimotor, and postural-control processes. Under this account, stimulation-related speeding of central processing could reduce the temporal delay between tasks and thereby attenuate interference, even when the structural bottleneck remains intact.

Target also matters for domain-specific effects. Among the included studies, the most frequently-investigated cognitive domains were executive function (including task-switching, inhibitory control, and cognitive flexibility), divided attention, and working memory (short-term information storage and manipulation). DLPFC stimulation may primarily influence executive allocation and attentional control, reducing prefrontal resource consumption for task monitoring and improving neural efficiency, yet its effects on working memory are modest unless combined with concurrent cognitive training (Jor'dan et al., 2022; Khanmohammadi et al., 2025b; Hertrich et al., 2021; Cieslik et al., 2015). M1 stimulation produces indirect cognitive benefits mainly by enhancing locomotor automaticity, thereby freeing up executive-attentional resources rather than directly altering high-order cognitive strategies (Khanmohammadi et al., 2025a, 2025b; Miterko et al., 2019). In contrast to the former two, cerebellar stimulation shows limited effects on executive function and attention allocation, but exhibits specific potential for modulating working-memory maintenance as well as temporal prediction and error-based motor adaptation (Khanmohammadi et al., 2015, 2025c). This target-dependence is supported by a recent randomized, double-blind crossover study in older adults that directly compared high-definition tDCS over left DLPFC, M1, and the cerebellum and reported significant improvements in dual-task gait parameters across all three targets, yet found no clear superiority of any single site, suggesting overlapping but non-identical mechanisms (Khanmohammadi et al., 2025b). Such findings reinforce that our pooled, target-heterogeneous effect estimates may conceal domain- and site-specific benefits that future separately-targeted trials will need to resolve.

The absence of a clear STV effect deserves particular attention. STV reflects stride-to-stride regulation and is often interpreted as an index of gait automaticity and dynamic control rather than simply walking speed (Porciuncula et al., 2025). Individual included trials reported improvements in selected variability or DTC measures, but the healthy pooled estimates crossed the null and heterogeneity was high. Differences from apparently positive single-study findings may arise because study-level consolidation removes pseudo-replication from multiple active arms, because serial subtraction, verbal, and other cognitive tasks impose different executive loads, and because STV is sensitive to trial duration, number of strides, walking surface, and algorithmic definition (Yona et al., 2025). Neurophysiologically, prefrontal stimulation may improve attention allocation without consistently normalizing basal-ganglia, cerebellar, and spinal timing processes that generate stride-to-stride regularity (Monaghan et al., 2025; Wei et al., 2025). The present result therefore suggests outcome specificity rather than a contradiction of the resource-allocation hypothesis.

Cadence and stride length were not meta-analysable in healthy participants, and their clinical estimates were inconclusive. These parameters depend on locomotor pattern generation, lower-limb biomechanics, disease-related constraints, and the co-intervention, so a prefrontal or motor-cortical excitability change may not translate uniformly into spatial or temporal gait adaptation (Liu et al., 2021). Stride time also could not be pooled by population, while DTC of stride time was heterogeneous and null in healthy adults. These findings differ from reports of short-term improvement in selected spatiotemporal measures, but the difference is plausibly explained by small samples, heterogeneous stimulation targets, task-priority instructions, and our correction for dependent multi-arm comparisons.

TUG and standing-balance outcomes also require cautious interpretation. TUG combines transfers, straight walking, turning, and sitting, creating several potential bottlenecks, while a concurrent cognitive task adds executive and attentional demands (Fudickar et al., 2022; Poole et al., 2022). The present evidence remains insufficient to identify a reliable tDCS effect on this composite outcome. Two Parkinson’s disease studies did not show a clear pooled effect, and only one healthy study was available. Standing sway depends on multisensory integration, ankle and hip strategies, and nonlinear postural dynamics (Iqbal, 2011; Ivanenko and Gurfinkel, 2018). Although the mixed-population forest plots suggested reductions in DTC of sway speed, sway area, and DTC of sway area, each estimate was based on one healthy and one functionally limited clinical study. These statistically significant findings should therefore be regarded as exploratory and cannot be generalized across populations or interpreted as evidence of fall prevention.

Taken together, the results are most consistent with a selective, short-term influence of tDCS on attentional allocation during dual-task gait rather than a generalized normalization of locomotor or postural control. The robust direction of gait-speed DTC across corr assumptions supports this interpretation, whereas the sensitivity of absolute gait speed and the null variability outcomes show that the evidence is not uniformly robust. Future trials should report paired crossover statistics, prespecify task priority, collect enough strides for variability analysis, assess motor and cognitive performance concurrently, stratify by diagnosis, and measure actual falls and longer-term outcomes.

## 5 Limitations

This review has several limitations. Most pooled outcomes included only two to six studies with small samples. Crossover trials required an assumed within-participant correlation, and multiple effects within a study required an assumed correlation during study-level consolidation; alternative assumptions could change precision. Although this approach avoids double counting shared sham conditions, individual-participant data would permit more exact modelling. Other limitations include heterogeneous populations, stimulation protocols and DT paradigms; incomplete cognitive-task and adverse-event reporting; limited follow-up; and too few studies for reliable publication-bias tests.

## 6 Conclusion

In healthy older adults, tDCS may improve gait speed and reduce DTC of gait speed under dual-task conditions. The DTC finding was stable across corr assumptions, whereas the gait-speed finding was not significant at corr = 0.25 and should be regarded as assumption-sensitive. Evidence for variability outcomes was inconclusive, and several other outcomes could not be pooled within healthy participants. Clinical populations should not be assumed to share the same effect. Evidence regarding actual falls, long-term benefit, and safety remains insufficient. Future studies should adopt multicenter large-sample designs as well as individual-participant-data meta-analysis to mitigate limitations arising from intervention heterogeneity and correlation-based assumptions. Moreover, independent stratified validation is required for distinct clinical subgroups, and findings should not be generalized to other populations. Extended follow-up periods incorporating hard endpoints such as falls are warranted. In accordance with the CONSORT guidelines, adverse events and comprehensive cognitive outcomes should be systematically reported to strengthen evidence for clinical translation and safety profiles of this intervention.

## Data Availability

The datasets presented in this study can be found in online repositories. The names of the repository/repositories and accession number(s) can be found in the article/Supplementary material. Requests to access these primary datasets should be directed to the corresponding authors of those original publications. For inquiries regarding the meta-analytic data presented in this manuscript, please contact the corresponding author: jinghuaqian891273@126.com.

## References

[ref1] Abedanzadeh R. Alboghebish S. Barati P. (2021). The effect of transcranial direct current stimulation of dorsolateral prefrontal cortex on performing a sequential dual task: a randomized experimental study. Psicol Reflex Crit 34:30. doi: 10.1186/s41155-021-00195-8, 34626255 PMC8502187

[ref2] Atrsaei A. Corrà M. F. Dadashi F. Vila-Chã N. Maia L. Mariani B. . (2021). Gait speed in clinical and daily living assessments in Parkinson's disease patients: performance versus capacity. NPJ Parkinsons Dis. 7:24. doi: 10.1038/s41531-021-00171-0, 33674597 PMC7935857

[ref3] Baharlouei H. Sadeghi-demneh E. Mehravar M. Manzari P. Shaterzadeh Yazdi M. J. Joghataei M. T. . (2020). Comparison of transcranial direct current stimulation of the primary motor cortex and cerebellum on static balance in older adults. Iran. Red Cresc. Med. J. 22:e96259. doi: 10.5812/ircmj.96259

[ref4] Barolo J. Mitchell D. Wade F. E. (2026). Effect of cognitive and motor dual-task on stability and variability of walking in younger and older adults. Exp. Gerontol. 214:113035. doi: 10.1016/j.exger.2026.113035, 41534647

[ref5] Beauchet O. Annweiler C. Dubost V. Allali G. Kressig R. W. Bridenbaugh S. . (2009). Stops walking when talking: a predictor of falls in older adults? Eur. J. Neurol. 16, 786–795. doi: 10.1111/j.1468-1331.2009.02612.x, 19473368

[ref6] Beck Jepsen D. Robinson K. Ogliari G. Montero-Odasso M. Kamkar N. Ryg J. . (2022). Predicting falls in older adults: an umbrella review of instruments assessing gait, balance, and functional mobility. BMC Geriatr. 22:615. doi: 10.1186/s12877-022-03271-5, 35879666 PMC9310405

[ref7] Beurskens R. Bock O. (2012). Age-related deficits of dual-task walking: a review. Neural Plast. 2012:131608. doi: 10.1155/2012/131608, 22848845 PMC3403123

[ref8] Bortone I. Sardone R. Lampignano L. Castellana F. Zupo R. Lozupone M. . (2021). How gait influences frailty models and health-related outcomes in clinical-based and population-based studies: a systematic review. J. Cachexia. Sarcopenia Muscle 12, 274–297. doi: 10.1002/jcsm.12667, 33590975 PMC8061366

[ref9] Cieslik E. C. Mueller V. I. Eickhoff C. R. Langner R. Eickhoff S. B. (2015). Three key regions for supervisory attentional control: evidence from neuroimaging meta-analyses. Neurosci. Biobehav. Rev. 48, 22–34. doi: 10.1016/j.neubiorev.2014.11.003, 25446951 PMC4272620

[ref10] Clark D. J. (2015). Automaticity of walking: functional significance, mechanisms, measurement and rehabilitation strategies. Front. Hum. Neurosci. 9:246. doi: 10.3389/fnhum.2015.00246 PMC4419715 25999838

[ref11] Folstein M. F. Folstein S. E. McHugh P. R. (1975). "Mini-mental state". A practical method for grading the cognitive state of patients for the clinician. J. Psychiatr. Res. 12, 189–198. doi: 10.1016/0022-3956(75)90026-6, 1202204

[ref12] Fudickar S. Pauls A. Lau S. Hellmers S. Gebel K. Diekmann R. . (2022). Measurement system for unsupervised standardized assessments of timed up and go test and 5 times chair rise test in community settings—a usability study. Sensors 22:731. doi: 10.3390/s22030731, 35161478 PMC8840449

[ref13] Hertrich I. Dietrich S. Blum C. Ackermann H. (2021). The role of the dorsolateral prefrontal cortex for speech and language processing. Front. Hum. Neurosci. 15:645209. doi: 10.3389/fnhum.2021.645209 PMC8165195 34079444

[ref14] Higgins CJ Cumpston M Li T Page MJ Welch VA. Cochrane Handbook for Systematic Reviews of Interventions version 6.3 (updated February 2022) (2022) Available online at: https://www.cochrane.org/authors/handbooks-and-manuals/handbook (Accessed October 21, 2025).

[ref15] Higgins J. P. Thompson S. G. Deeks J. J. Altman D. G. (2003). Measuring inconsistency in meta-analyses. BMJ 327, 557–560. doi: 10.1136/bmj.327.7414.557, 12958120 PMC192859

[ref16] Hupfeld K. E. Geraghty J. M. McGregor H. R. Hass C. J. Pasternak O. Seidler R. D. (2022). Differential relationships between brain structure and dual task walking in young and older adults. Front. Aging Neurosci. 14:809281. doi: 10.3389/fnagi.2022.809281 PMC8963788 35360214

[ref17] Iqbal K, Editor mechanisms and models of postural stability and control (2011). Annual International Conference of the IEEE Engineering in Medicine and Biology Society. 10.1109/IEMBS.2011.6091931 22256156

[ref18] Ivanenko Y. Gurfinkel V. S. (2018). Human postural control. Front. Neurosci. 12:171. doi: 10.3389/fnins.2018.00171 PMC5869197 29615859

[ref19] Jor'dan A. J. Bernad-Elazari H. Mirelman A. Gouskova N. A. Lo O. Y. Hausdorff J. M. . (2022). Transcranial direct current stimulation may reduce prefrontal recruitment during dual task walking in functionally limited older adults - a pilot study. Front. Aging Neurosci. 14:843122. doi: 10.3389/fnagi.2022.843122 PMC8963782 35360209

[ref20] Kalkhoran Z. B. Khanmohammadi R. (2025). The effects of aquatic therapy combined with transcranial direct current stimulation (tDCS) on proprioception and gait speed in older adults with knee osteoarthritis: an eight-week randomized sham-controlled trial. BMC Geriatr. 25:676. doi: 10.1186/s12877-025-06253-5, 40898048 PMC12403502

[ref21] Khanmohammadi R. Inanlu M. Manesh V. (2025a). Region-specific cognitive effects of HD-tDCS in older adults: M1, DLPFC, and cerebellum. Behav. Brain Res. 486:115571. doi: 10.1016/j.bbr.2025.115571, 40174444

[ref22] Khanmohammadi R. Inanlu M. Manesh V. R. (2025b). Effects of HD-tDCS targeting prefrontal cortex, cerebellum, and motor cortex on dual-task gait in older adults. Sci. Rep. 15:31899. doi: 10.1038/s41598-025-17655-1, 40883499 PMC12397365

[ref23] Khanmohammadi R. Talebian S. Hadian M. R. Olyaei G. Bagheri H. (2015). Preparatory postural adjustments during gait initiation in healthy younger and older adults: neurophysiological and biomechanical aspects. Brain Res. 1629, 240–249. doi: 10.1016/j.brainres.2015.09.039, 26453832

[ref24] Koppelmans V. Hoogendam Y. Y. Hirsiger S. Mérillat S. Jäncke L. Seidler R. D. (2017). Regional cerebellar volumetric correlates of manual motor and cognitive function. Brain Struct. Funct. 222, 1929–1944. doi: 10.1007/s00429-016-1317-7, 27699480

[ref25] Lara K. E. A. Linares J. C. C. Montilla J. A. P. Román P. Á. L. (2024). Factors influencing gait performance in older adults in a dual-task paradigm. Geroscience. 46, 3071–3083. doi: 10.1007/s11357-023-01052-5, 38190081 PMC11009214

[ref26] Lau C. I. Liu M. N. Cheng F. Y. Wang H. C. Walsh V. Liao Y. Y. (2024). Can transcranial direct current stimulation combined with interactive computerized cognitive training boost cognition and gait performance in older adults with mild cognitive impairment? A randomized controlled trial. J. Neuroeng. Rehabil. 21:26. doi: 10.1186/s12984-024-01313-0, 38365761 PMC10874043

[ref27] Lee J. Han S. W. (2020). Visual search proceeds concurrently during the attentional blink and response selection bottleneck. Atten. Percept. Psychophys. 82, 2893–2908. doi: 10.3758/s13414-020-02047-6, 32406002

[ref28] Lefaucheur J. P. Antal A. Ayache S. S. Benninger D. H. Brunelin J. Cogiamanian F. . (2017). Evidence-based guidelines on the therapeutic use of transcranial direct current stimulation (tDCS). Clin. Neurophysiol. 128, 56–92. doi: 10.1016/j.clinph.2016.10.087, 27866120

[ref29] Liao Y. Y. Liu M. N. Wang H. C. Walsh V. Lau C. I. (2021). Combining transcranial direct current stimulation with tai chi to improve dual-task gait performance in older adults with mild cognitive impairment: a randomized controlled trial. Front. Aging Neurosci. 13:13. doi: 10.3389/fnagi.2021.766649, 34966268 PMC8710779

[ref30] Liu X. Liu H. Liu Z. Rao J. Wang J. Wang P. . (2021). Transcranial direct current stimulation for Parkinson's disease: a systematic review and meta-analysis. Front. Aging Neurosci. 13:746797. doi: 10.3389/fnagi.2021.746797 PMC8584149 34776931

[ref31] Liu S. Rosso A. L. Baillargeon E. M. Weinstein A. M. Rosano C. Torres-Oviedo G. (2024). Novel attentional gait index reveals a cognitive ability-related decline in gait automaticity during dual-task walking. Front. Aging Neurosci. 15:1283376. doi: 10.3389/fnagi.2023.1283376 PMC10808635 38274986

[ref32] Ljubisavljevic M. R. Oommen J. Filipovic S. Bjekic J. Szolics M. Nagelkerke N. (2019). Effects of tDCS of dorsolateral prefrontal cortex on dual-task performance involving manual dexterity and cognitive task in healthy older adults. Front. Aging Neurosci. 11:144. doi: 10.3389/fnagi.2019.00144, 31275139 PMC6592113

[ref33] Lundh A. Gøtzsche P. C. (2008). Recommendations by Cochrane review groups for assessment of the risk of bias in studies. BMC Med. Res. Methodol. 8:22. doi: 10.1186/1471-2288-8-22, 18426565 PMC2375895

[ref34] Luo J. Su L. Carter S. J. Ndeke J. M. Hendryx M. (2023). Optimal objective measurement of physical function and its predictive capacity for mortality among community-dwelling older women. Geriatr Gerontol Int 23, 715–721. doi: 10.1111/ggi.14654, 37650477 PMC11503693

[ref35] Manor B. Zhou J. Harrison R. Lo O. Y. Travison T. G. Hausdorff J. M. . (2018). Transcranial direct current stimulation may improve cognitive-motor function in functionally limited older adults. Neurorehabil. Neural Repair 32, 788–798. doi: 10.1177/1545968318792616, 30132389 PMC6143414

[ref36] Manor B. Zhou J. Jor'dan A. Zhang J. Fang J. Pascual-Leone A. (2016). Reduction of dual-task costs by noninvasive modulation of prefrontal activity in healthy elders. J. Cogn. Neurosci. 28, 275–281. doi: 10.1162/jocn_a_00897, 26488591 PMC4751581

[ref37] Mishra R. K. Thrasher A. T. (2022). Effect of concurrent transcranial direct current stimulation on instrumented timed up and go task performance in people with Parkinson's disease: a double-blind and cross-over study. J. Clin. Neurosci. 100, 184–191. doi: 10.1016/j.jocn.2022.04.029, 35487026

[ref38] Miterko L. N. Baker K. B. Beckinghausen J. Bradnam L. V. Cheng M. Y. Cooperrider J. . (2019). Consensus paper: experimental Neurostimulation of the cerebellum. Cerebellum 18, 1064–1097. doi: 10.1007/s12311-019-01041-5, 31165428 PMC6867990

[ref39] Miura H. Ono Y. Suzuki T. Ogihara Y. Imai Y. Watanabe A. . (2024). Regional brain activity and neural network changes in cognitive-motor dual-task interference: a functional near-infrared spectroscopy study. NeuroImage 297:120714. doi: 10.1016/j.neuroimage.2024.120714 38950665

[ref40] Monaghan A. S. Takla T. Ofori E. Peterson D. S. Wu W. Fritz N. E. . (2025). The neural contributions to reactive balance control: a scoping review of EEG, fNIRS, MRI, and PET studies. Brain Sci. 15:1330. doi: 10.3390/brainsci15121330, 41440126 PMC12730421

[ref41] Montero-Odasso M. Verghese J. Beauchet O. Hausdorff J. M. (2012). Gait and cognition: a complementary approach to understanding brain function and the risk of falling. J. Am. Geriatr. Soc. 60, 2127–2136. doi: 10.1111/j.1532-5415.2012.04209.x, 23110433 PMC3498517

[ref42] Nasreddine Z. S. Phillips N. A. Bédirian V. Charbonneau S. Whitehead V. Collin I. . (2005). The Montreal cognitive assessment, MoCA: a brief screening tool for mild cognitive impairment. J. Am. Geriatr. Soc. 53, 695–699. doi: 10.1111/j.1532-5415.2005.53221.x, 15817019

[ref43] Nitsche M. A. Paulus W. (2000). Excitability changes induced in the human motor cortex by weak transcranial direct current stimulation. J. Physiol. 527, 633–639. doi: 10.1111/j.1469-7793.2000.t01-1-00633.x PMC2270099 10990547

[ref44] Orcioli-Silva D. Islam A. Baker M. R. Gobbi L. T. B. Rochester L. Pantall A. (2021). Bi-anodal transcranial direct current stimulation combined with treadmill walking decreases motor cortical activity in young and older adults. Front. Aging Neurosci. 13:739998. doi: 10.3389/fnagi.2021.739998, 34924993 PMC8681021

[ref45] Paas Oliveros L. K. Pieczykolan A. Pläschke R. N. Eickhoff S. B. Langner R. (2023). Response-code conflict in dual-task interference and its modulation by age. Psychol. Res. 87, 260–280. doi: 10.1007/s00426-021-01639-7, 35122495 PMC9352817

[ref46] Page M. J. McKenzie J. E. Bossuyt P. M. Boutron I. Hoffmann T. C. Mulrow C. D. . (2020). The PRISMA 2020 statement: an updated guideline for reporting systematic reviews. BMJ 372:n71. doi: 10.1136/bmj.n71, 33782057 PMC8005924

[ref47] Page M. J. Moher D. Bossuyt P. M. Boutron I. Hoffmann T. C. Mulrow C. D. . (2021). PRISMA 2020 explanation and elaboration: updated guidance and exemplars for reporting systematic reviews. BMJ 372:n160. doi: 10.1136/bmj.n160, 33781993 PMC8005925

[ref48] Pino-Esteban A. Megía-García Á. Martín-Caro Álvarez D. Beltran-Alacreu H. Avendaño-Coy J. Gómez-Soriano J. . (2021). Can transcranial direct current stimulation enhance functionality in older adults? A systematic review. J. Clin. Med. 10:2981. doi: 10.3390/jcm10132981, 34279465 PMC8268136

[ref49] Poole V. N. Dawe R. J. Lamar M. Esterman M. Barnes L. Leurgans S. E. . (2022). Dividing attention during the timed up and go enhances associations of several subtask performances with MCI and cognition. PLoS One 17:e0269398. doi: 10.1371/journal.pone.0269398, 35921260 PMC9348700

[ref50] Porciuncula F. Cavanaugh J. T. Zajac J. Wendel N. Baker T. Arumukhom Revi D. . (2025). Amplifying walking activity in Parkinson’s disease through autonomous music-based rhythmic auditory stimulation: randomized controlled trial. NPJ Parkinsons Dis 11:100. doi: 10.1038/s41531-025-00952-x, 40301366 PMC12041193

[ref51] Rafiee Manesh V. Inanlu M. Yekaninejad M. S. Khanmohammadi R. (2024). The effect of different types of cognitive tasks on postural sway fluctuations in older and younger adults: a nonlinear study. J. Bodyw. Mov. Ther. 37, 63–69. doi: 10.1016/j.jbmt.2023.11.014, 38432843

[ref52] Ramírez F. Gutiérrez M. (2021). Dual-task gait as a predictive tool for cognitive impairment in older adults: a systematic review. Front. Aging Neurosci. 13:769462. doi: 10.3389/fnagi.2021.769462, 35002676 PMC8740025

[ref53] Rodrigues N. O. Vidal Bravalhieri A. A. Moraes T. P. Barros J. A. Ansai J. H. Christofoletti G. (2023). Efficacy of transcranial direct current stimulation (tDCS) on cognition, anxiety, and mobility in community-dwelling older individuals: a controlled clinical trial. Brain Sci. 13:1614. doi: 10.3390/brainsci13121614, 38137062 PMC10741841

[ref54] Rowland R. S. Jenkinson N. Chiou S.-Y. (2021). Age-related differences in corticospinal excitability and anticipatory postural adjustments of the trunk. Front. Aging Neurosci. 13:718784. doi: 10.3389/fnagi.2021.718784 PMC8416077 34483887

[ref55] Saraiva M. Fernandes O. J. Vilas-Boas J. P. Castro M. A. (2022). Standing posture in motor and cognitive dual-tasks during smartphone use: linear and nonlinear analysis of postural control. Euro. J. Invest. Health Psychol. Educ. 12, 1021–1033. doi: 10.3390/ejihpe12080073, 36005222 PMC9407520

[ref56] Saraiva M. Vilas-Boas J. P. Fernandes O. J. Castro M. A. (2023). Effects of motor task difficulty on postural control complexity during dual tasks in young adults: a nonlinear approach. Sensors 23:628. doi: 10.3390/s23020628, 36679423 PMC9866022

[ref57] Schabrun S. M. Lamont R. M. Brauer S. G. (2016). Transcranial direct current stimulation to enhance dual-task gait training in Parkinson's disease: a pilot RCT. PLoS One 11:e0158497. doi: 10.1371/journal.pone.0158497, 27359338 PMC4928827

[ref58] Schneider N. Dagan M. Katz R. Thumm P. C. Brozgol M. Giladi N. . (2021). Combining transcranial direct current stimulation with a motor-cognitive task: the impact on dual-task walking costs in older adults. J. Neuroeng. Rehabil. 18:23. doi: 10.1186/s12984-021-00826-2, 33526043 PMC7852224

[ref59] Smith J. A. Fisher B. E. (2018). Anticipatory postural adjustments and spatial organization of motor cortex: evidence of adaptive compensations in healthy older adults. J. Neurophysiol. 120, 2796–2805. doi: 10.1152/jn.00428.2018, 30281380

[ref60] Stedman M. R. Curtin F. Elbourne D. R. Kesselheim A. S. Brookhart M. A. (2011). Meta-analyses involving cross-over trials: methodological issues. Int. J. Epidemiol. 40, 1732–1734. doi: 10.1093/ije/dyp345 20026595

[ref61] Studenski S. Perera S. Patel K. Rosano C. Faulkner K. Inzitari M. . (2011). Gait speed and survival in older adults. JAMA 305, 50–58. doi: 10.1001/jama.2010.1923, 21205966 PMC3080184

[ref62] Swank C. Mehta J. Criminger C. (2016). Transcranial direct current stimulation lessens dual task cost in people with Parkinson's disease. Neurosci. Lett. 626, 1–5. doi: 10.1016/j.neulet.2016.05.010, 27181509

[ref63] Tsang C. S.-L. Wang S. Miller T. Pang M. Y.-C. (2022). Degree and pattern of dual-task interference during walking vary with component tasks in people after stroke: a systematic review. J. Physiother. 68, 26–36. doi: 10.1016/j.jphys.2021.12.009, 34953757

[ref64] Watanabe K. Funahashi S. (2018). Toward an understanding of the neural mechanisms underlying dual-task performance: contribution of comparative approaches using animal models. Neurosci. Biobehav. Rev. 84, 12–28. doi: 10.1016/j.neubiorev.2017.08.008, 28851576

[ref65] Wei Y. Chen P. Zhan J. Yin L. Yu Z. Wang L. (2025). The effect of transcranial direct current stimulation on static and dynamic posture control in the elderly: a systematic review and meta-analysis. Front. Aging Neurosci. 17:1645962. doi: 10.3389/fnagi.2025.1645962 PMC12380813 40881197

[ref66] Wei P. Zou T. Lv Z. Fan Y. (2020). Functional MRI reveals locomotion-control neural circuits in human brainstem. Brain Sci. 10:757. doi: 10.3390/brainsci10100757, 33092164 PMC7589833

[ref67] World Health Organization Falls:Key Facts (2021). Available online at: https://www.who.int/news-room/fact-sheets/detail/falls (Accessed January 10, 2026).

[ref68] Yogev-Seligmann G. Hausdorff J. M. Giladi N. (2008). The role of executive function and attention in gait. Mov. Disord. 23, 329–342. doi: 10.1002/mds.21720, 18058946 PMC2535903

[ref69] Yona T. Peskin B. Fischer A. (2025). The COMPWALK-ACL: a dataset of multi-pace IMU gait kinematics in adolescents, adults, and ACL injured patients. Sci. Data 13:4. doi: 10.1038/s41597-025-06307-8, 41366255 PMC12764956

[ref70] Yun S. J. Hyun S. E. Lee W. H. Oh B. M. Seo H. G. (2025). Comparison of stimulation sites enhancing dual-task performance using transcranial direct current stimulation in Parkinson's disease. NPJ Parkinsons Dis. 11:19. doi: 10.1038/s41531-025-00869-5, 39827184 PMC11742881

[ref71] Zhou J. Manor B. Yu W. Lo O. Y. Gouskova N. Salvador R. . (2021). Targeted tDCS mitigates dual-task costs to gait and balance in older adults. Ann. Neurol. 90, 428–439. doi: 10.1002/ana.26156, 34216034 PMC8434977

[ref72] Zhou T. Straub E. Kiesel A. Gehring D. Granacher U. Haslbauer A. . (2026). Functional reserve mitigates cognitive-motor dual-task costs in older adults: insights from age, cohort, and behavioural strategies. Eur. Rev. Aging Phys. Act. 23:24. doi: 10.1186/s11556-026-00423-z, 42350948 PMC13326005

[ref73] Zhou D. Zhou J. Chen H. Manor B. Lin J. Zhang J. (2015). Effects of transcranial direct current stimulation (tDCS) on multiscale complexity of dual-task postural control in older adults. Exp. Brain Res. 233, 2401–2409. doi: 10.1007/s00221-015-4310-0, 25963755 PMC4746714

