# Supplementary material for: Effects of transcranial direct current stimulation on gait and balance under dual-task conditions in older adults: a systematic review and meta-analysis
Source: Front Aging Neurosci. 2026 Sep 28;18:1924261. doi: 10.3389/fnagi.2026.1924261 (PMC13655153; doi:10.3389/fnagi.2026.1924261)
Supplement: Supplementary file 1 [file Data_Sheet_1.DOCX]

Supplementary Material

# 1 Supplementary Search Strategy

## 1.1 PubMed：

### 1.1.1 **MESH：**

Transcranial Direct Current Stimulation

### 1.1.2 Free Words：

**tDCS:** “Anodal Stimulation Transcranial Direct Current Stimulation” OR “Anodal Stimulation tDCS*” OR “Cathodal Stimulation tDCS*” OR “Cathodal Stimulation Transcranial Direct Current Stimulation” OR “Repetitive Transcranial Electrical Stimulation” OR “ tDCS” OR “Transcranial Alternating Current Stimulation” OR “Transcranial Electrical Stimulation*” OR “Transcranial Random Noise Stimulation” OR “neuromodulation” OR “non-invasive brain stimulation”

**Older adults**：“aged” OR “aging” OR “elderly” OR “older adult*” OR “elder adult*” OR “old” OR “older”

**Dual Task：**“dual task performance” OR “dual task” OR “dual task test” OR “dual task paradigm” OR “Gait” OR “dual task gait” OR “dual task walking” OR “posture control” OR “postural control” OR “dual task postural control” OR “cognitive function” OR “cognition” OR “dual task cognition” OR “motor cognitive”

## 1.2 Embase and Cochrane Library

### 1.2.1 MESH：

Transcranial Direct Current Stimulation

### 1.2.2 Free Words：

**tDCS:** (‘Anodal Stimulation Transcranial Direct Current Stimulation’ OR ‘Anodal Stimulation tDCS*’ OR ‘Cathodal Stimulation tDCS*’ OR ‘Repetitive Transcranial Electrical Stimulation’ OR ‘ tDCS’ OR ‘Transcranial Alternating Current Stimulation’ OR ‘Transcranial Electrical Stimulation*’ OR ‘Transcranial Random Noise Stimulation’ OR ‘neuromodulation’ OR ‘non-invasive brain stimulation’):ti,ab,kw

**Older adults:** (‘aged’ OR ‘aging’ OR ‘elderly’ OR ‘older adult*’ OR ‘elder adult*’ OR ‘old’ OR ‘older’):ti,ab,kw

**Dual Task:** (‘dual task performance’ OR ‘dual task’ OR ‘dual task test’ OR ‘dual task paradigm’ OR ‘Gait’ OR ‘dual task gait’ OR ‘dual task walking’ OR ‘posture control’ OR ‘postural control’ OR ‘dual task postural control’ OR ‘cognitive function’ OR ‘cognition’ OR ‘dual task cognition’ OR ‘motor cognitive’):ti,ab,kw

## 1.3 Web of Science

**tDCS:** ((((((((((TS=(Transcranial Direct Current Stimulation)) OR TS=(Anodal Stimulation Transcranial Direct Current Stimulation)) OR TS=(Anodal Stimulation tDCS*)) OR TS=(Cathodal Stimulation tDCS*)) OR TS=(Repetitive Transcranial Electrical Stimulation)) OR TS=(tDCS)) OR TS=(Transcranial Alternating Current Stimulation)) OR TS=(Transcranial Electrical Stimulation*)) OR TS=(Transcranial Random Noise Stimulation)) OR TS=(neuromodulation)) OR TS=(non-invasive brain stimulation)

**Older adults:** ((((((TS=(aged)) OR TS=(aging)) OR TS=(elderly)) OR TS=(older adult*)) OR TS=(elder adult*)) OR TS=(old)) OR TS=(older)

**Dual Task:** (((((((((((((TS=(dual task performance)) OR TS=(dual task)) OR TS=(dual task test)) OR TS=(dual task paradigm)) OR TS=(Gait)) OR TS=(dual task gait)) OR TS=(dual task walking)) OR TS=(posture control)) OR TS=(postural control)) OR TS=(dual task postural control)) OR TS=(cognitive function)) OR TS=(cognition)) OR TS=(dual task cognition)) OR TS=(motor cognitive).

# 2 Supplementary Table S1. Correlation sensitivity analyses: healthy primary population

| **Outcome** | **corr** | **k** | **g** | **95% CI** | **P** | **I² (%)** |
| --- | --- | --- | --- | --- | --- | --- |
| Gait speed | 0.25 | 2 | 0.203 | -0.020 to 0.426 | 0.074 | 0.0 |
| Gait speed | 0.5 | 2 | 0.200 | 0.017 to 0.383 | 0.032 | 0.0 |
| Gait speed | 0.75 | 2 | 0.215 | 0.022 to 0.408 | 0.029 | 42.5 |
| DTC of gait speed | 0.25 | 2 | -0.381 | -0.609 to -0.153 | 0.001 | 0.0 |
| DTC of gait speed | 0.5 | 2 | -0.368 | -0.557 to -0.179 | <0.001 | 0.0 |
| DTC of gait speed | 0.75 | 2 | -0.332 | -0.470 to -0.193 | <0.001 | 0.0 |
| DTC of stride time | 0.25 | 2 | 0.060 | -0.660 to 0.780 | 0.871 | 81.4 |
| DTC of stride time | 0.5 | 2 | 0.032 | -0.610 to 0.675 | 0.921 | 84.0 |
| DTC of stride time | 0.75 | 2 | -0.017 | -0.503 to 0.469 | 0.946 | 85.1 |
| STV | 0.25 | 2 | -0.167 | -0.506 to 0.172 | 0.334 | 46.9 |
| STV | 0.5 | 2 | -0.181 | -0.525 to 0.164 | 0.305 | 63.6 |
| STV | 0.75 | 2 | -0.193 | -0.539 to 0.154 | 0.277 | 80.4 |
| DTC of STV | 0.25 | 4 | 0.528 | -0.517 to 1.573 | 0.322 | 93.9 |
| DTC of STV | 0.5 | 4 | 0.515 | -0.441 to 1.471 | 0.291 | 94.5 |
| DTC of STV | 0.75 | 4 | 0.488 | -0.310 to 1.285 | 0.231 | 95.0 |
| DTC of SLV | 0.25 | 2 | -0.011 | -1.093 to 1.071 | 0.984 | 78.3 |
| DTC of SLV | 0.5 | 2 | -0.015 | -1.087 to 1.057 | 0.978 | 79.3 |
| DTC of SLV | 0.75 | 2 | -0.014 | -1.065 to 1.036 | 0.979 | 80.0 |

# 3 Supplementary Table S2. Correlation sensitivity analyses: exploratory mixed-population estimates

| **Outcome** | **corr** | **k** | **g** | **95% CI** | **P** | **I² (%)** |
| --- | --- | --- | --- | --- | --- | --- |
| Gait speed | 0.25 | 6 | 0.055 | -0.228 to 0.338 | 0.702 | 38.0 |
| Gait speed | 0.5 | 6 | 0.071 | -0.199 to 0.340 | 0.607 | 41.9 |
| Gait speed | 0.75 | 6 | 0.092 | -0.154 to 0.338 | 0.464 | 48.4 |
| DTC of gait speed | 0.25 | 5 | -0.370 | -0.577 to -0.163 | <0.001 | 0.0 |
| DTC of gait speed | 0.5 | 5 | -0.362 | -0.538 to -0.185 | <0.001 | 0.0 |
| DTC of gait speed | 0.75 | 5 | -0.331 | -0.464 to -0.197 | <0.001 | 0.0 |
| Cadence | 0.25 | 3 | 0.332 | -0.457 to 1.121 | 0.410 | 63.0 |
| Cadence | 0.5 | 3 | 0.332 | -0.457 to 1.121 | 0.410 | 63.0 |
| Cadence | 0.75 | 3 | 0.332 | -0.457 to 1.121 | 0.410 | 63.0 |
| Stride length | 0.25 | 3 | 0.301 | -0.051 to 0.654 | 0.094 | 0.0 |
| Stride length | 0.5 | 3 | 0.298 | -0.010 to 0.606 | 0.058 | 0.0 |
| Stride length | 0.75 | 3 | 0.292 | 0.054 to 0.531 | 0.016 | 0.0 |
| Stride time | 0.25 | 2 | -0.194 | -0.586 to 0.197 | 0.331 | 0.0 |
| Stride time | 0.5 | 2 | -0.215 | -0.548 to 0.117 | 0.204 | 0.0 |
| Stride time | 0.75 | 2 | -0.239 | -0.487 to 0.010 | 0.060 | 0.0 |
| DTC of stride time | 0.25 | 3 | 0.000 | -0.535 to 0.536 | 0.999 | 65.0 |
| DTC of stride time | 0.5 | 3 | -0.016 | -0.514 to 0.482 | 0.950 | 69.3 |
| DTC of stride time | 0.75 | 3 | -0.046 | -0.446 to 0.354 | 0.821 | 71.0 |
| STV | 0.25 | 2 | -0.167 | -0.506 to 0.172 | 0.334 | 46.9 |
| STV | 0.5 | 2 | -0.181 | -0.525 to 0.164 | 0.305 | 63.6 |
| STV | 0.75 | 2 | -0.193 | -0.539 to 0.154 | 0.277 | 80.4 |
| DTC of STV | 0.25 | 5 | 0.383 | -0.493 to 1.259 | 0.391 | 91.9 |
| DTC of STV | 0.5 | 5 | 0.376 | -0.439 to 1.191 | 0.366 | 92.6 |
| DTC of STV | 0.75 | 5 | 0.360 | -0.336 to 1.057 | 0.311 | 93.4 |
| TUG time | 0.25 | 3 | -0.128 | -0.460 to 0.204 | 0.449 | 0.0 |
| TUG time | 0.5 | 3 | -0.153 | -0.483 to 0.177 | 0.364 | 17.5 |
| TUG time | 0.75 | 3 | -0.184 | -0.526 to 0.158 | 0.291 | 44.0 |
| DTC of SLV | 0.25 | 2 | -0.011 | -1.093 to 1.071 | 0.984 | 78.3 |
| DTC of SLV | 0.5 | 2 | -0.015 | -1.087 to 1.057 | 0.978 | 79.3 |
| DTC of SLV | 0.75 | 2 | -0.014 | -1.065 to 1.036 | 0.979 | 80.0 |
| Sway speed | 0.25 | 2 | -0.241 | -0.694 to 0.213 | 0.298 | 31.1 |
| Sway speed | 0.5 | 2 | -0.244 | -0.678 to 0.189 | 0.270 | 31.1 |
| Sway speed | 0.75 | 2 | -0.246 | -0.633 to 0.141 | 0.213 | 27.5 |
| DTC of sway speed | 0.25 | 2 | -0.272 | -0.524 to -0.020 | 0.034 | 0.0 |
| DTC of sway speed | 0.5 | 2 | -0.271 | -0.482 to -0.060 | 0.012 | 0.0 |
| DTC of sway speed | 0.75 | 2 | -0.248 | -0.404 to -0.092 | 0.002 | 0.0 |
| Sway area | 0.25 | 2 | -0.227 | -0.479 to 0.024 | 0.076 | 0.0 |
| Sway area | 0.5 | 2 | -0.221 | -0.431 to -0.011 | 0.039 | 0.0 |
| Sway area | 0.75 | 2 | -0.195 | -0.350 to -0.040 | 0.013 | 0.0 |
| DTC of sway area | 0.25 | 2 | -0.306 | -0.562 to -0.051 | 0.019 | 0.0 |
| DTC of sway area | 0.5 | 2 | -0.288 | -0.503 to -0.073 | 0.009 | 0.0 |
| DTC of sway area | 0.75 | 2 | -0.229 | -0.390 to -0.068 | 0.005 | 0.0 |

# 4 Supplementary Table S3. Summary of findings: Effects of tDCS on dual‑task gait parameters in healthy older adults

| **Certainty assessment** | | | | | | | **№ of patients** | | **Effect** | | **Certainty** | **Importance** |
| --- | --- | --- | --- | --- | --- | --- | --- | --- | --- | --- | --- | --- |
| **№ of studies** | **Study design** | **Risk of bias** | **Inconsistency** | **Indirectness** | **Imprecision** | **Other considerations** | **tDCS** | **sham tDCS** | **Relative (95% CI)** | **Absolute (95% CI)** |  |  |
| **Dual‑task gait speed (assessed with: Instrumented gait mat (m/s))** | | | | | | | | | | | | |
| 2 | randomised trials | not serious^a^ | not serious | not serious | serious^b^ | none | 76 | 76 | - | SMD **0.2 SD higher** (0.017 higher to 0.383 higher) | ⨁⨁⨁◯ Moderate^a,b^ | CRITICAL |
| **Dual-task cost of gait speed (assessed with: Calculated as percentage change in gait speed from single-task to dual-task walking)** | | | | | | | | | | | | |
| 2 | randomised trials | not serious^a^ | not serious | not serious | serious^b^ | none | 76 | 76 | - | SMD **0.368 SD lower** (0.557 lower to 0.179 lower) | ⨁⨁⨁◯ Moderate^a,b^ | CRITICAL |
| **Dual-task cost of stride time (assessed with: Dual‑task cost calculated from stride‑time under single‑task and dual‑task walking)** | | | | | | | | | | | | |
| 2 | randomised trials | serious^c^ | serious^d^ | not serious | serious^e^ | none | 44 | 44 | - | SMD **0.032 SD higher** (0.61 lower to 0.675 higher) | ⨁◯◯◯ Very low^c,d,e^ | IMPORTANT |
| **Stride time variability (assessed with: Instrumented walkway (e.g., GAITRite) or wearable inertial sensors; calculated as the standard deviation (SD) or coefficient of variation (CV) of stride-to-stride time during walking.)** | | | | | | | | | | | | |
| 2 | randomised trials | not serious^a^ | serious^f^ | not serious | serious^g^ | none | 76 | 76 | - | SMD **0.181 SD lower** (0.525 lower to 0.164 higher) | ⨁⨁◯◯ Low^a,f,g^ | IMPORTANT |
| **Dual-task cost of stride time variability (assessed with: gait parameters recorded via electronic walkway or wearable sensors; DTC calculated as [(single‑task coefficient of variation – dual‑task coefficient of variation) / single‑task coefficient of variation] × 100%)** | | | | | | | | | | | | |
| 4 | randomised trials | serious^h^ | serious^i^ | not serious | serious^j^ | none | 111 | 109 | - | SMD **0.515 SD higher** (0.441 lower to 1.471 higher) | ⨁◯◯◯ Very low^h,i,j^ | IMPORTANT |
| **Dual-task cost of stride length variability (assessed with: Calculated as [(single-task coefficient of variation − dual-task coefficient of variation) / single-task coefficient of variation] × 100%)** | | | | | | | | | | | | |
| 2 | randomised trials | serious^k^ | serious^l^ | not serious | serious^m^ | none | 29 | 27 | - | SMD **0.015 SD lower** (1.087 lower to 1.057 lower) | ⨁◯◯◯ Very low^k,l,m^ |  |

**CI:** confidence interval; **SMD:** standardised mean difference

## Explanations

a. One crossover trial was rated as some concerns for risk of bias according to RoB 2.0, while the other was judged as low risk of bias. None of the included studies were assessed as high risk of bias.

b. Only two included trials with small total sample size (n = 76). The optimal information size criterion was not met.

c. One contributing crossover RCT (Schneider N et al. 2021) was judged overall high risk of bias in RoB‑2, mainly driven by high risk for missing outcome data (D3). The second crossover RCT (Khanmohammadi R et al. 2025) raised some concerns in the domain of missing outcome data (D3).

d. I² = 84.0%; point estimates in opposite directions across studies (Schneider et al. showed large positive effects, while Khanmohammadi et al. showed negative/null effects).

e. Total sample size (n = 44) from only two small crossover RCTs was well below the optimal information size. The 95% confidence interval was very wide and crossed the line of no‑effect, encompassing both clinically important benefit and clinically important harm.

f. Moderate‑substantial statistical heterogeneity (I² = 63.6%). Effect directions were consistent across the two studies, yet effect magnitudes differed materially. Methodological differences existed: online‑versus‑offline tDCS paradigms, different electrode systems (conventional multi‑target tDCS versus high‑definition tDCS), and potential differences in gait‑measurement hardware and STV computation. Sources of heterogeneity could not be further explored given only two included studies.

g. Total n = 76 (Zhou n=57; Khanmohammadi n=19) below OIS; wide 95% CI crossing null, covering both clinically important benefit and harm.

h. Downgraded by one level because only one of four studies (≈48% weight) was at low risk of bias; one study was at high risk and two had some concerns regarding randomization, missing data, or selective reporting.

i. Downgraded by one level for serious inconsistency due to very high statistical heterogeneity (I² = 94.5%, P < 0.001). Sensitivity analysis confirmed that one single influential study reversed the overall effect estimate when removed. Substantial between‑trial differences in dual‑task paradigms and tDCS montage contributed to this inconsistency.

j. Downgraded by one level for serious imprecision. The total sample size was small(N = 119, below the conventional OIS threshold), Moreover, the 95% confidence interval for the pooled estimate was wide (−0.441 to 1.471) and crossed the null, with the lower bound suggesting a moderate beneficial effect and the upper bound suggesting a substantial harmful effect, precluding a reliable estimate of the true effect.

k. Downgraded by one level for risk of bias, as both contributing studies were judged as having “some concerns” (Khanmohammadi 2025: randomization/carryover; Orcioli‑Silva 2021: selective reporting), with no low-risk study available to anchor the evidence body.

l. Downgraded by one level for serious inconsistency: substantial heterogeneity (I² = 79.3%).

m. Downgraded by one level for serious imprecision. The pooled estimate was based on only 37 participants, far below the conventional OIS threshold of 400 for continuous outcomes. The 95% confidence interval was extremely wide (−1.087 to 1.057), crossed the null value (0), and encompassed both a large beneficial effect and a moderate harmful effect, precluding any reliable inference about the true effect.

# 5 Supplementary Table S4. Summary of findings: Effects of tDCS on dual‑task gait parameters in clinical older adults

| **Certainty assessment** | | | | | | | **№ of patients** | | **Effect** | | **Certainty** | **Importance** |
| --- | --- | --- | --- | --- | --- | --- | --- | --- | --- | --- | --- | --- |
| **№ of studies** | **Study design** | **Risk of bias** | **Inconsistency** | **Indirectness** | **Imprecision** | **Other considerations** | **tDCS** | **sham tDCS** | **Relative (95% CI)** | **Absolute (95% CI)** |  |  |
| **Dual-task of gait speed (assessed with: Instrumented gait mat (m/s))** | | | | | | | | | | | | |
| 4 | randomised trials | serious^a^ | not serious | not serious | serious^b^ | none | 46 | 45 | - | SMD **0.158 SD lower** (0.607 lower to 0.29 higher) | ⨁⨁◯◯ Low^a,b^ | CRITICAL |
| **Dual-task cost of gait speed (assessed with: Calculated as percentage change in gait speed from single-task to dual-task walking)** | | | | | | | | | | | | |
| 3 | randomised trials | not serious | not serious | not serious | serious^c^ | none | 30 | 29 | - | SMD **0.319 SD lower** (0.815 lower to 0.177 higher) | ⨁⨁⨁◯ Moderate^c^ | CRITICAL |
| **Duan-task of cadence (assessed with: spatiotemporal gait parameters recorded via electronic walkway or wearable inertial sensors; cadence measured as steps per minute under dual‑task conditions)** | | | | | | | | | | | | |
| 3 | randomised trials | not serious | serious^d^ | not serious | serious^e^ | none | 29 | 28 | - | SMD **0.332 SD higher** (0.457 lower to 1.121 higher) | ⨁⨁◯◯ Low^d,e^ | IMPORTANT |
| **Dual -task of stride length (assessed with: Dual‑task gait analysis (stride length via instrumented walkway or IMU sensors))** | | | | | | | | | | | | |
| 2 | randomised trials | not serious | not serious | not serious | serious^f^ | none | 21 | 20 | - | SMD **0.328 SD higher** (0.267 lower to 0.922 higher) | ⨁⨁⨁◯ Moderate^f^ | IMPORTANT |
| **Dual-task of Timed Up and Go (TUG) test time (assessed with: Timed Up and Go test under dual‑task conditions (performed concurrently with a cognitive task, e.g., serial subtraction or carrying a cup))** | | | | | | | | | | | | |
| 2 | randomised trials | serious^g^ | not serious | not serious | serious^h^ | none | 29 | 29 | - | SMD **0.241 SD lower** (0.641 lower to 0.159 higher) | ⨁⨁◯◯ Low^g,h^ | CRITICAL |

**CI:** confidence interval; **SMD:** standardised mean difference

## Explanations

a. Two ‘some concerns’ studies (Kalkhoran, Schabrun) accounted for 55% of the total weight, constituting the majority of evidence; downgraded one level for serious risk of bias.

b. Downgraded by one level. The pooled estimate was based on only 91 participants, far below the optimal information size for continuous outcomes; the 95% CI (−0.607 to 0.290) was wide and crossed the null, precluding a reliable estimate of the true effect.

c. Downgraded by one level. The pooled estimate was based on only 59 participants, far below the optimal information size for continuous outcomes; the 95% CI (−0.815 to 0.177) was wide and crossed the null, with the lower bound suggesting moderate benefit and the upper bound suggesting trivial harm, precluding a reliable estimate.

d. Downgraded by one level. Heterogeneity was substantial (I²=63.0%). Liao and Lau showed favourable trends (positive effects), whereas Schabrun showed unfavourable/null trends (negative effects), with limited overlap of confidence intervals, suggesting clinically meaningful inconsistency.

e. Downgraded by one level. The pooled estimate was based on only 57 participants, far below the optimal information size for continuous outcomes; the 95% CI (−0.457 to 1.121) was wide and crossed the null, with the lower bound suggesting harm and the upper bound suggesting substantial benefit, precluding a reliable estimate.

f. Downgraded by one level. The pooled estimate was based on only 41 participants, far below the optimal information size for continuous outcomes; the 95% CI (−0.267 to 0.922) crossed the null and was wide, with the lower bound suggesting trivial harm and the upper bound suggesting moderate benefit, precluding a reliable estimate.

g. Downgraded by one level for serious risk of bias; both included trials were rated as having some concerns overall.

h. Downgraded by one level. The pooled estimate was based on only 29 participants, far below the optimal information size for continuous outcomes; the 95% CI (−0.641 to 0.159) was wide and crossed the null, with the lower bound suggesting moderate benefit and the upper bound suggesting trivial harm, precluding a reliable estimate.

# 6 Supplementary Table S5. Comparison-level effect sizes before within-study consolidation

| **Outcome** | **Study** | **Active comparison** | **Comparator** | **Population** | **Design** | **Data** | **n A** | **n C** | **g** | **SE** | **Variance** | **95% CI** |
| --- | --- | --- | --- | --- | --- | --- | --- | --- | --- | --- | --- | --- |
| Gait speed | Zhou J et al.2021. | only DLPFC group(n=57) | Acti-sham group(n=57) | healthy | crossover | change | 57 | 57 | 0.1879 | 0.1319 | 0.01739 | -0.0706 to 0.4464 |
| Gait speed | Zhou J et al.2021. | only SM1 group(n=57) | Acti-sham group(n=57) | healthy | crossover | change | 57 | 57 | -0.0481 | 0.1308 | 0.01710 | -0.3044 to 0.2082 |
| Gait speed | Zhou J et al.2021. | L-DLPFC+ SM1 group(n=57) | Acti-sham group(n=57) | healthy | crossover | change | 57 | 57 | 0.3368 | 0.1345 | 0.01809 | 0.0732 to 0.6004 |
| Gait speed | Liao YY et al.2021. | a-tDCS + TC group(n=10) | sham + TC group(n=10) | clinical | parallel | change | 10 | 10 | 0.3071 | 0.4314 | 0.18608 | -0.5384 to 1.1526 |
| Gait speed | Lau CI et al.2024. | tDCS + ICCT group(n=11) | sham + ICCT group(n=10) | clinical | parallel | change | 11 | 10 | 0.2933 | 0.4221 | 0.17821 | -0.5341 to 1.1207 |
| Gait speed | Khanmohammadi R et al.2025. | L-DLPFC group(n=19) | Control group(n=19) | healthy | crossover | change | 19 | 19 | 0.4039 | 0.2298 | 0.05281 | -0.0465 to 0.8543 |
| Gait speed | Khanmohammadi R et al.2025. | M1 group(n=19) | Control group(n=19) | healthy | crossover | change | 19 | 19 | 0.3471 | 0.2272 | 0.05162 | -0.0982 to 0.7924 |
| Gait speed | Khanmohammadi R et al.2025. | Cerebellum group(n=19) | Control group(n=19) | healthy | crossover | change | 19 | 19 | 0.3016 | 0.2254 | 0.05081 | -0.1401 to 0.7434 |
| Gait speed | Kalkhoran ZB et al.2025. | Aquatic therapy+real tDCS group(n=17) | Aquatic therapy + sham tDCS group(n=17) | clinical | parallel | change | 17 | 17 | -0.0738 | 0.3350 | 0.11224 | -0.7304 to 0.5829 |
| Gait speed | Kalkhoran ZB et al.2025. | Real tDCS group(n=17) | Sham tDCS group(n=17) | clinical | parallel | change | 17 | 17 | -0.8167 | 0.3501 | 0.12258 | -1.5029 to -0.1304 |
| Gait speed | S. M. Schabrun et al.2016 | Active tDCS group(n=8) | Sham tDCS group(n=8) | clinical | parallel | change | 8 | 8 | -0.8824 | 0.5013 | 0.25128 | -1.8649 to 0.1001 |
| Gait speed | S. M. Schabrun et al.2016 | Active tDCS group(n=8) | Sham tDCS group(n=8) | clinical | parallel | change | 8 | 8 | -0.6545 | 0.4886 | 0.23877 | -1.6123 to 0.3032 |
| Gait speed | S. M. Schabrun et al.2016 | Active tDCS group(n=8) | Sham tDCS group(n=8) | clinical | parallel | change | 8 | 8 | -0.3763 | 0.4780 | 0.22853 | -1.3133 to 0.5607 |
| DTC of gait speed | Zhou J et al.2021. | only DLPFC group(n=57) | Acti-sham group(n=57) | healthy | crossover | change | 57 | 57 | -0.5674 | 0.1412 | 0.01995 | -0.8442 to -0.2905 |
| DTC of gait speed | Zhou J et al.2021. | only SM1 group(n=57) | Acti-sham group(n=57) | healthy | crossover | change | 57 | 57 | -0.1020 | 0.1310 | 0.01717 | -0.3588 to 0.1548 |
| DTC of gait speed | Zhou J et al.2021. | L-DLPFC+ SM1 group(n=57) | Acti-sham group(n=57) | healthy | crossover | change | 57 | 57 | -0.6187 | 0.1432 | 0.02049 | -0.8993 to -0.3381 |
| DTC of gait speed | Manor B et al.2018. | tDCS group(n=9) | sham tDCS group(n=9) | clinical | parallel | reported final | 9 | 9 | 0.1627 | 0.4499 | 0.20239 | -0.7191 to 1.0444 |
| DTC of gait speed | Liao YY et al.2021. | a-tDCS + TC group(n=10) | sham + TC group(n=10) | clinical | parallel | change | 10 | 10 | -0.4401 | 0.4346 | 0.18883 | -1.2918 to 0.4117 |
| DTC of gait speed | Lau CI et al.2024. | tDCS + ICCT group(n=11) | sham + ICCT group(n=10) | clinical | parallel | change | 11 | 10 | -0.6430 | 0.4322 | 0.18682 | -1.4901 to 0.2042 |
| DTC of gait speed | Khanmohammadi R et al.2025. | L-DLPFC group(n=19) | Control group(n=19) | healthy | crossover | change | 19 | 19 | -0.2400 | 0.2233 | 0.04988 | -0.6777 to 0.1977 |
| DTC of gait speed | Khanmohammadi R et al.2025. | M1 group(n=19) | Control group(n=19) | healthy | crossover | change | 19 | 19 | -0.6018 | 0.2415 | 0.05834 | -1.0753 to -0.1284 |
| DTC of gait speed | Khanmohammadi R et al.2025. | Cerebellum group(n=19) | Control group(n=19) | healthy | crossover | change | 19 | 19 | -0.2300 | 0.2230 | 0.04975 | -0.6672 to 0.2071 |
| Cadence | Liao YY et al.2021. | a-tDCS + TC group(n=10) | sham + TC group(n=10) | clinical | parallel | change | 10 | 10 | 0.7293 | 0.4452 | 0.19823 | -0.1434 to 1.6019 |
| Cadence | Lau CI et al.2024. | tDCS + ICCT group(n=11) | sham + ICCT group(n=10) | clinical | parallel | change | 11 | 10 | 0.7553 | 0.4370 | 0.19095 | -0.1012 to 1.6118 |
| Cadence | S. M. Schabrun et al.2016 | Active tDCS group(n=8) | Sham tDCS group(n=8) | clinical | parallel | change | 8 | 8 | -0.5044 | 0.4822 | 0.23256 | -1.4496 to 0.4408 |
| Cadence | S. M. Schabrun et al.2016 | Active tDCS group(n=8) | Sham tDCS group(n=8) | clinical | parallel | change | 8 | 8 | -0.6319 | 0.4876 | 0.23773 | -1.5875 to 0.3238 |
| Cadence | S. M. Schabrun et al.2016 | Active tDCS group(n=8) | Sham tDCS group(n=8) | clinical | parallel | change | 8 | 8 | -0.1668 | 0.4738 | 0.22446 | -1.0954 to 0.7618 |
| Stride length | Liao YY et al.2021. | a-tDCS + TC group(n=10) | sham + TC group(n=10) | clinical | parallel | change | 10 | 10 | 0.0616 | 0.4284 | 0.18356 | -0.7781 to 0.9014 |
| Stride length | Lau CI et al.2024. | tDCS + ICCT group(n=11) | sham + ICCT group(n=10) | clinical | parallel | change | 11 | 10 | 0.5978 | 0.4305 | 0.18535 | -0.2460 to 1.4416 |
| Stride length | Khanmohammadi R et al.2025. | L-DLPFC group(n=19) | Control group(n=19) | healthy | crossover | change | 19 | 19 | 0.3090 | 0.2257 | 0.05093 | -0.1333 to 0.7514 |
| Stride length | Khanmohammadi R et al.2025. | M1 group(n=19) | Control group(n=19) | healthy | crossover | change | 19 | 19 | 0.3149 | 0.2259 | 0.05103 | -0.1279 to 0.7577 |
| Stride length | Khanmohammadi R et al.2025. | Cerebellum group(n=19) | Control group(n=19) | healthy | crossover | change | 19 | 19 | 0.2381 | 0.2233 | 0.04985 | -0.1995 to 0.6757 |
| Stride time | Manor B et al.2018. | tDCS group(n=9) | sham tDCS group(n=9) | clinical | parallel | reported final | 9 | 9 | 0.1267 | 0.4495 | 0.20206 | -0.7543 to 1.0078 |
| Stride time | Khanmohammadi R et al.2025. | L-DLPFC group(n=19) | Control group(n=19) | healthy | crossover | change | 19 | 19 | -0.2618 | 0.2240 | 0.05018 | -0.7009 to 0.1772 |
| Stride time | Khanmohammadi R et al.2025. | M1 group(n=19) | Control group(n=19) | healthy | crossover | change | 19 | 19 | -0.3470 | 0.2272 | 0.05162 | -0.7923 to 0.0983 |
| Stride time | Khanmohammadi R et al.2025. | Cerebellum group(n=19) | Control group(n=19) | healthy | crossover | change | 19 | 19 | -0.2153 | 0.2226 | 0.04957 | -0.6516 to 0.2211 |
| DTC of stride time | Schneider N et al.2021. | tDCS + walking group(n=25) | sham + walking group(n=25) | healthy | crossover | change | 25 | 25 | 1.1260 | 0.2528 | 0.06393 | 0.6305 to 1.6216 |
| DTC of stride time | Schneider N et al.2021. | tDCS + seated group(n=25) | sham + walking group(n=25) | healthy | crossover | change | 25 | 25 | 0.1041 | 0.1943 | 0.03774 | -0.2767 to 0.4848 |
| DTC of stride time | Manor B et al.2018. | tDCS group(n=9) | sham tDCS group(n=9) | clinical | parallel | reported final | 9 | 9 | -0.2149 | 0.4506 | 0.20300 | -1.0980 to 0.6682 |
| DTC of stride time | Khanmohammadi R et al.2025. | L-DLPFC group(n=19) | Control group(n=19) | healthy | crossover | change | 19 | 19 | -0.1892 | 0.2220 | 0.04927 | -0.6243 to 0.2458 |
| DTC of stride time | Khanmohammadi R et al.2025. | M1 group(n=19) | Control group(n=19) | healthy | crossover | change | 19 | 19 | -0.6322 | 0.2437 | 0.05938 | -1.1098 to -0.1546 |
| DTC of stride time | Khanmohammadi R et al.2025. | Cerebellum group(n=19) | Control group(n=19) | healthy | crossover | change | 19 | 19 | -0.1956 | 0.2221 | 0.04934 | -0.6310 to 0.2398 |
| STV | Zhou J et al.2021. | only DLPFC group(n=57) | Acti-sham group(n=57) | healthy | crossover | change | 57 | 57 | 0.0601 | 0.1308 | 0.01711 | -0.1963 to 0.3165 |
| STV | Zhou J et al.2021. | only SM1 group(n=57) | Acti-sham group(n=57) | healthy | crossover | change | 57 | 57 | 0.0000 | 0.1307 | 0.01708 | -0.2561 to 0.2561 |
| STV | Zhou J et al.2021. | L-DLPFC+ SM1 group(n=57) | Acti-sham group(n=57) | healthy | crossover | change | 57 | 57 | -0.1686 | 0.1316 | 0.01733 | -0.4266 to 0.0894 |
| STV | Khanmohammadi R et al.2025. | L-DLPFC group(n=19) | Control group(n=19) | healthy | crossover | change | 19 | 19 | -0.4348 | 0.2314 | 0.05353 | -0.8882 to 0.0187 |
| STV | Khanmohammadi R et al.2025. | M1 group(n=19) | Control group(n=19) | healthy | crossover | change | 19 | 19 | -0.3072 | 0.2256 | 0.05090 | -0.7494 to 0.1350 |
| STV | Khanmohammadi R et al.2025. | Cerebellum group(n=19) | Control group(n=19) | healthy | crossover | change | 19 | 19 | -0.4479 | 0.2321 | 0.05385 | -0.9028 to 0.0069 |
| DTC of STV | Zhou J et al.2021. | only DLPFC group(n=57) | Acti-sham group(n=57) | healthy | crossover | change | 57 | 57 | -0.4037 | 0.1361 | 0.01853 | -0.6705 to -0.1369 |
| DTC of STV | Zhou J et al.2021. | only SM1 group(n=57) | Acti-sham group(n=57) | healthy | crossover | change | 57 | 57 | -0.1093 | 0.1311 | 0.01718 | -0.3663 to 0.1476 |
| DTC of STV | Zhou J et al.2021. | L-DLPFC+ SM1 group(n=57) | Acti-sham group(n=57) | healthy | crossover | change | 57 | 57 | -0.2521 | 0.1328 | 0.01764 | -0.5124 to 0.0083 |
| DTC of STV | Schneider N et al.2021. | tDCS + walking group(n=25) | sham + walking group(n=25) | healthy | crossover | change | 25 | 25 | 3.9703 | 0.6049 | 0.36591 | 2.7846 to 5.1559 |
| DTC of STV | Schneider N et al.2021. | tDCS + seated group(n=25) | sham + walking group(n=25) | healthy | crossover | change | 25 | 25 | 2.5230 | 0.4125 | 0.17013 | 1.7146 to 3.3315 |
| DTC of STV | Orcioli-Silva D et al.2021. | Active tDCS (n = 10) | Sham tDCS (n = 8) | healthy | parallel | change | 10 | 8 | 0.1645 | 0.4527 | 0.20493 | -0.7227 to 1.0518 |
| DTC of STV | Manor B et al.2018. | tDCS group(n=9) | sham tDCS group(n=9) | clinical | parallel | reported final | 9 | 9 | -0.2149 | 0.4506 | 0.20300 | -1.0980 to 0.6682 |
| DTC of STV | Khanmohammadi R et al.2025. | L-DLPFC group(n=19) | Control group(n=19) | healthy | crossover | change | 19 | 19 | -0.3512 | 0.2274 | 0.05170 | -0.7969 to 0.0945 |
| DTC of STV | Khanmohammadi R et al.2025. | M1 group(n=19) | Control group(n=19) | healthy | crossover | change | 19 | 19 | -0.3504 | 0.2273 | 0.05169 | -0.7960 to 0.0952 |
| DTC of STV | Khanmohammadi R et al.2025. | Cerebellum group(n=19) | Control group(n=19) | healthy | crossover | change | 19 | 19 | -0.3965 | 0.2294 | 0.05264 | -0.8462 to 0.0532 |
| TUG time | Rodrigues NO et al.2023. | tDCS group(n=14) | tDCS-Placebo group(n=13) | healthy | parallel | change | 14 | 13 | 0.1946 | 0.3745 | 0.14025 | -0.5395 to 0.9286 |
| TUG time | Seo Jung Yun et al.2025 | M1 group(n=19) | Sham tDCS(n=19) | clinical | crossover | change | 19 | 19 | -0.1486 | 0.2211 | 0.04889 | -0.5820 to 0.2848 |
| TUG time | Seo Jung Yun et al.2025 | L-DLPFC group(n=19) | Sham tDCS(n=19) | clinical | crossover | change | 19 | 19 | -0.0837 | 0.2202 | 0.04847 | -0.5152 to 0.3479 |
| TUG time | Seo Jung Yun et al.2025 | vmPFC(n=19) | Sham tDCS(n=19) | clinical | crossover | change | 19 | 19 | -0.0685 | 0.2200 | 0.04841 | -0.4997 to 0.3627 |
| TUG time | C. Swank et al.2016 | Active group(n=10) | Sham group(n=10) | clinical | crossover | final | 10 | 10 | -0.5364 | 0.3156 | 0.09957 | -1.1548 to 0.0821 |
| DTC of SLV | Orcioli-Silva D et al.2021. | Active tDCS (n = 10) | Sham tDCS (n = 8) | healthy | parallel | change | 10 | 8 | 0.6190 | 0.4648 | 0.21606 | -0.2920 to 1.5301 |
| DTC of SLV | Khanmohammadi R et al.2025. | L-DLPFC group(n=19) | Control group(n=19) | healthy | crossover | change | 19 | 19 | -0.3830 | 0.2288 | 0.05235 | -0.8314 to 0.0655 |
| DTC of SLV | Khanmohammadi R et al.2025. | M1 group(n=19) | Control group(n=19) | healthy | crossover | change | 19 | 19 | -0.7191 | 0.2503 | 0.06264 | -1.2097 to -0.2286 |
| DTC of SLV | Khanmohammadi R et al.2025. | Cerebellum group(n=19) | Control group(n=19) | healthy | crossover | change | 19 | 19 | -0.4422 | 0.2318 | 0.05371 | -0.8964 to 0.0121 |
| Sway speed | Zhou J et al.2021. | only DLPFC group(n=57) | Acti-sham group(n=57) | healthy | crossover | change | 57 | 57 | -0.5208 | 0.1396 | 0.01950 | -0.7944 to -0.2471 |
| Sway speed | Zhou J et al.2021. | only SM1 group(n=57) | Acti-sham group(n=57) | healthy | crossover | change | 57 | 57 | -0.1358 | 0.1313 | 0.01724 | -0.3931 to 0.1216 |
| Sway speed | Zhou J et al.2021. | L-DLPFC+ SM1 group(n=57) | Acti-sham group(n=57) | healthy | crossover | change | 57 | 57 | -0.4729 | 0.1381 | 0.01907 | -0.7436 to -0.2023 |
| Sway speed | Manor B et al.2018. | tDCS group(n=9) | sham tDCS group(n=9) | clinical | parallel | reported final | 9 | 9 | 0.2056 | 0.4504 | 0.20288 | -0.6772 to 1.0884 |
| DTC of sway speed | Zhou J et al.2021. | only DLPFC group(n=57) | Acti-sham group(n=57) | healthy | crossover | change | 57 | 57 | -0.4776 | 0.1382 | 0.01911 | -0.7486 to -0.2067 |
| DTC of sway speed | Zhou J et al.2021. | only SM1 group(n=57) | Acti-sham group(n=57) | healthy | crossover | change | 57 | 57 | 0.0156 | 0.1307 | 0.01708 | -0.2405 to 0.2718 |
| DTC of sway speed | Zhou J et al.2021. | L-DLPFC+ SM1 group(n=57) | Acti-sham group(n=57) | healthy | crossover | change | 57 | 57 | -0.5285 | 0.1399 | 0.01957 | -0.8027 to -0.2543 |
| DTC of sway speed | Manor B et al.2018. | tDCS group(n=9) | sham tDCS group(n=9) | clinical | parallel | reported final | 9 | 9 | 0.0956 | 0.4493 | 0.20185 | -0.7850 to 0.9762 |
| Sway area | Zhou J et al.2021. | only DLPFC group(n=57) | Acti-sham group(n=57) | healthy | crossover | change | 57 | 57 | -0.3648 | 0.1351 | 0.01826 | -0.6297 to -0.0999 |
| Sway area | Zhou J et al.2021. | only SM1 group(n=57) | Acti-sham group(n=57) | healthy | crossover | change | 57 | 57 | 0.1027 | 0.1310 | 0.01717 | -0.1542 to 0.3595 |
| Sway area | Zhou J et al.2021. | L-DLPFC+ SM1 group(n=57) | Acti-sham group(n=57) | healthy | crossover | change | 57 | 57 | -0.5420 | 0.1404 | 0.01970 | -0.8171 to -0.2670 |
| Sway area | Manor B et al.2018. | tDCS group(n=9) | sham tDCS group(n=9) | clinical | parallel | reported final | 9 | 9 | -0.0464 | 0.4490 | 0.20163 | -0.9265 to 0.8337 |
| DTC of sway area | Zhou J et al.2021. | only DLPFC group(n=57) | Acti-sham group(n=57) | healthy | crossover | change | 57 | 57 | -0.5604 | 0.1410 | 0.01988 | -0.8368 to -0.2841 |
| DTC of sway area | Zhou J et al.2021. | only SM1 group(n=57) | Acti-sham group(n=57) | healthy | crossover | change | 57 | 57 | 0.1099 | 0.1311 | 0.01718 | -0.1471 to 0.3668 |
| DTC of sway area | Zhou J et al.2021. | L-DLPFC+ SM1 group(n=57) | Acti-sham group(n=57) | healthy | crossover | change | 57 | 57 | -0.6988 | 0.1464 | 0.02144 | -0.9858 to -0.4119 |
| DTC of sway area | Manor B et al.2018. | tDCS group(n=9) | sham tDCS group(n=9) | clinical | parallel | reported final | 9 | 9 | -0.0754 | 0.4492 | 0.20174 | -0.9557 to 0.8050 |
